# Fnr (EtrA) acts as a fine-tuning regulator of anaerobic metabolism in *Shewanella oneidensis *MR-1

**DOI:** 10.1186/1471-2180-11-64

**Published:** 2011-03-30

**Authors:** Claribel Cruz-García, Alison E Murray, Jorge LM Rodrigues, Jeffrey A Gralnick, Lee Ann McCue, Margaret F Romine, Frank E Löffler, James M Tiedje

**Affiliations:** 1 Center for Microbial Ecology, Michigan State University, East Lansing, Michigan 48824-1325, USA; 2 Department of Crop and Soil Sciences, Michigan State University, East Lansing, Michigan 48824-1325, USA; 3 Department of Microbiology and Molecular Genetics, Michigan State University, East Lansing, Michigan 48824-1325, USA; 4 Department of Biology, University of Texas at Arlington, Arlington, Texas 76019, USA; 5 Department of Microbiology, University of Minnesota, St. Paul, MN 55108, USA; 6 Pacific Northwest National Laboratory, Richland, Washington 99352, USA; 7 Department of Microbiology, University of Tennessee, Knoxville, MN 37996, USA; 8 Department of Civil and Environmental Engineering, University of Tennessee, Knoxville, MN 37996, USA; 9 Oak Ridge National Laboratory, Oak Ridge, TN 37831, USA; 10 Georgia Institute of Technology, School of Civil and Environmental Engineering, 311 Ferst Drive, Atlanta, GA 30332-0512, USA; 11 Division of Earth and Ecosystem Sciences, Desert Research Institute, Reno, NV 89512, USA

## Abstract

**Background:**

EtrA in *Shewanella oneidensis *MR-1, a model organism for study of adaptation to varied redox niches, shares 73.6% and 50.8% amino acid sequence identity with the oxygen-sensing regulators Fnr in *E. coli *and Anr in *Pseudomonas aeruginosa*, respectively; however, its regulatory role of anaerobic metabolism in *Shewanella *spp. is complex and not well understood.

**Results:**

The expression of the *nap *genes, *nrfA, cymA *and *hcp *was significantly reduced in *etrA *deletion mutant EtrA7-1; however, limited anaerobic growth and nitrate reduction occurred, suggesting that multiple regulators control nitrate reduction in this strain. Dimethyl sulfoxide (DMSO) and fumarate reductase gene expression was down-regulated at least 2-fold in the mutant, which, showed lower or no reduction of these electron acceptors when compared to the wild type, suggesting both respiratory pathways are under EtrA control. Transcript analysis further suggested a role of EtrA in prophage activation and down-regulation of genes implicated in aerobic metabolism.

**Conclusion:**

In contrast to previous studies that attributed a minor regulatory role to EtrA in *Shewanella *spp., this study demonstrates that EtrA acts as a global transcriptional regulator and, in conjunction with other regulators, fine-tunes the expression of genes involved in anaerobic metabolism in *S. oneidensis *strain MR-1. Transcriptomic and sequence analyses of the genes differentially expressed showed that those mostly affected by the mutation belonged to the "Energy metabolism" category, while stress-related genes were indirectly regulated in the mutant possibly as a result of a secondary perturbation (e.g. oxidative stress, starvation). We also conclude based on sequence, physiological and expression analyses that this regulator is more appropriately termed Fnr and recommend this descriptor be used in future publications.

## Background

Due to its respiratory versatility, *Shewanella oneidensis *strain MR-1 serves as a model organism for studying the regulation of aerobic and anaerobic growth [1-3]. In contrast to *Escherichia coli*, the regulatory systems that control transcription of genes responsible for different respiratory processes are poorly understood in environmentally relevant *Shewanella *spp. [4-7]. In *E. coli*, the transition from aerobic to anaerobic metabolism is primarily regulated by Fnr (fumarate and nitrate reduction regulator) and by the two-component regulatory system ArcAB (aerobic respiration control) [8-11]. A gene expression study in *E. coli *K12 indicated that one-third of its 4,290 genes were differentially expressed during aerobic versus anaerobic growth [12]. Among the differentially expressed genes, 712 (49%) genes were directly or indirectly affected by Fnr. Fnr possesses a [4Fe-4S]^2+ ^cluster that acts as an oxygen sensory domain [13]. Fnr in its active dimeric form binds to target DNA sequences inducing or repressing transcription [14,15]. Under aerobic conditions, or when oxygen levels increase, an Fe^2+ ^atom in the [4Fe-4S]^2+ ^cluster is oxidized resulting in the formation of a [2Fe-2S]^2+ ^cluster via a [3Fe-4S]^1+ ^intermediate. This oxidation causes a conformation change in Fnr, thus altering its affinity to DNA and regulatory control of transcription [14,15].

Studies with strain MR-1 mutants have identified three important regulators of anaerobic metabolism: EtrA (electron transport regulator protein), ArcA and CRP (cyclic AMP receptor protein) [4,5,16-19]. Sequence alignment of the protein encoded by *etrA *reveal that the four cysteine residues that form the [4Fe-4S]^2+ ^cluster in Fnr are conserved in EtrA [16]. In a gene replacement study, *etrA *of strain MR-1 restored wild type physiology of an *E. coli fnr *deletion mutant [16]. EtrA shares 73.6% and 50.8% of amino acid sequence identity with Fnr in *E. coli *and Anr (arginine deaminase and nitrate reductase anaerobic regulator) in *Pseudomonas aeruginosa*, respectively. This high degree of similarity suggests that EtrA has a regulatory function in MR-1, possibly by sensing oxygen. Despite the lack of physiological evidence to support a regulatory role of EtrA in the anaerobic metabolism of strain MR-1 [7], a gene expression study using a partial microarray (691 ORFs) of the strain MR-1's genome suggested involvement of EtrA in the regulation of the transcription of genes associated with aerobic and anaerobic metabolism [6]. Growth experiments with an *etrA *deletion mutant in *S. oneidensis *strain DSP10 (a spontaneous rifampicin resistant mutant of MR-1) implicated EtrA in the regulation of genes related to aerobic and anaerobic metabolism, similar to what has been observed for Fnr in *E. coli *[12,20]. Unfortunately, the implications of these findings cannot be interpreted unambiguously since the rifampicin resistance of strain DSP10 influences electron transport [21].

To examine the regulatory role of EtrA in strain MR-1 in more detail, we generated an *etrA *knockout mutant EtrA7-1 in a wild type background. Growth and phenotypic characterization of this mutant combined with a whole genome transcriptome analysis confirms that EtrA regulates nitrate and fumarate reduction, plus provides experimental evidence for its positive regulatory role in DMSO reduction. Our genome-wide expression analysis shows differential expression of 612 genes for which sequence analysis recognized a EtrA motif for 72 of the operons encoding 118 genes, suggesting that their regulation is via direct interaction of EtrA with its promoters. Most of these genes are associated with metabolic functions.

## Results

### Genotypic and phenotypic characterization of a Δ*etrA*::*loxP *mutant

**Figure 1:**
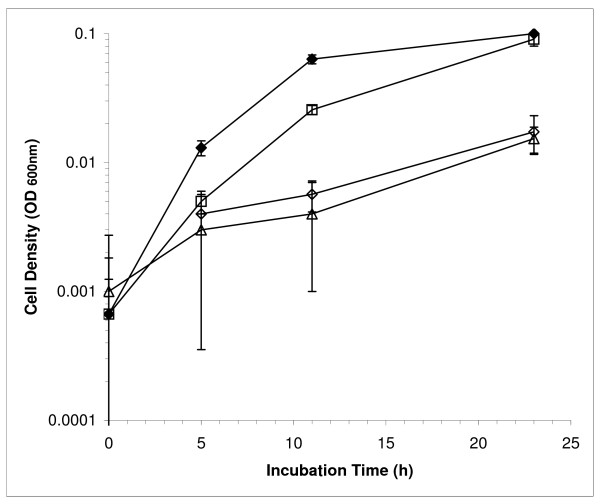
**Anaerobic growth of EtrA7-1 and the wild type strains on lactate and nitrate**. Wild type strain (closed diamonds), EtrA7-1 complement strain (open squares), EtrA7-1 (open diamonds) and EtrA7-1 harboring pCM62 (open triangles) served as a negative control. Data are means and SD from three independent cultures.

**Figure 2:**
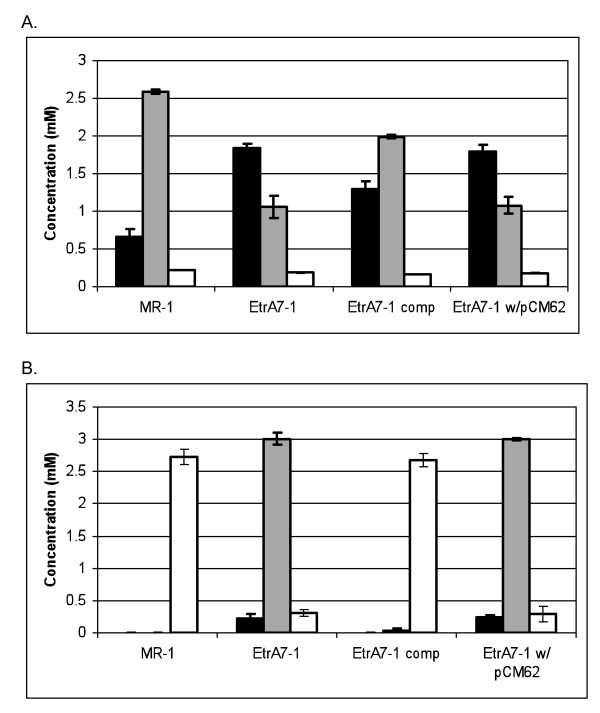
**Nitrate consumption and products formed during growth of the EtrA7-1 and wild type strains in Figure 1**. Samples were collected after 10 h (panel A) and 23 h (panel B) and analyzed for nitrate (black bar), nitrite (gray bar) and ammonium (white bar). Data are means and SD from three independent cultures.

The growth of the *etrA *knockout mutant EtrA7-1 with nitrate was significantly impaired as cultures reached a maximum OD_600 _of 0.02, at least 5-fold lower than the wild type strain (Figure 1). In addition, the doubling time for the mutant under these conditions was approximately 10 h compared to a doubling time of 2 h for the wild type. Plasmid pCCG03 carrying *etrA*, but not the parental pCM62 vector lacking *etrA*, restored near wild type growth to the EtrA7-1 mutant, which confirms that the observed phenotype was attributable to the deletion of *etrA*. After 10 h of incubation, nitrate was reduced in wild type and complemented EtrA7-1 cultures though less nitrate was reduced in the latter consistent with its slightly slower growth (Figure 2). After 23 h, both the wild type and EtrA7-1 complement had completely converted nitrate and nitrite to ammonia, while nitrite was the major product in cultures of the EtrA7-1 mutant and the control strain harboring pCM62 (Figure 2).

**Figure 3:**
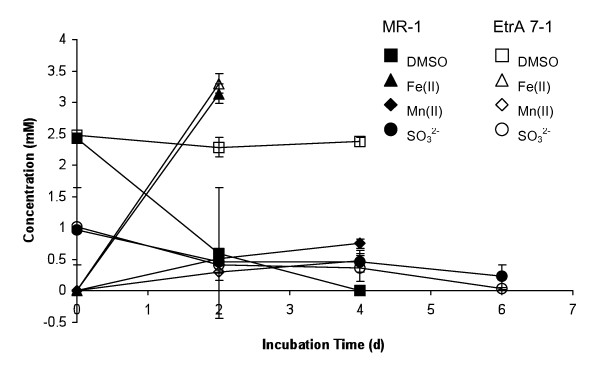
**Substrate consumption and intermediate production in anaerobic cultures of the wild type (closed symbols) and EtrA7-1 (open symbols) mutant strains grown with lactate and different electron acceptors**. DMSO consumption, squares; Fe(II) production, triangles; Mn(II) production, diamonds and sulfite consumption, circles. Data are means and SD from three independent cultures.

**Figure 4:**
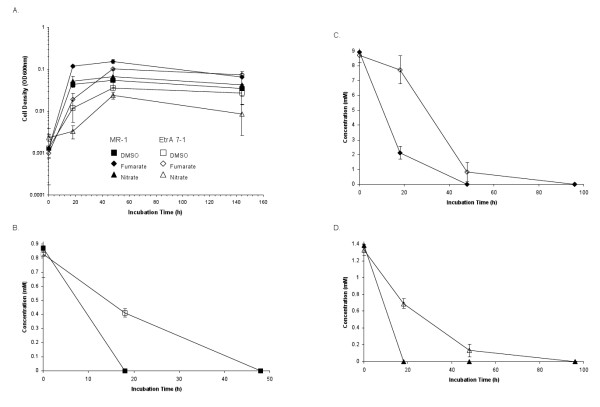
**Growth of the wild type (closed symbols) and Etra7-1 (open symbols) strains with pyruvate and the indicated electron acceptor**. (Panel A) DMSO consumption - squares (Panel B), fumarate consumption - diamonds (Panel C) and nitrate comsumption - triangles (Panel D). Data are means and SD from three independent cultures.

**Figure 5:**
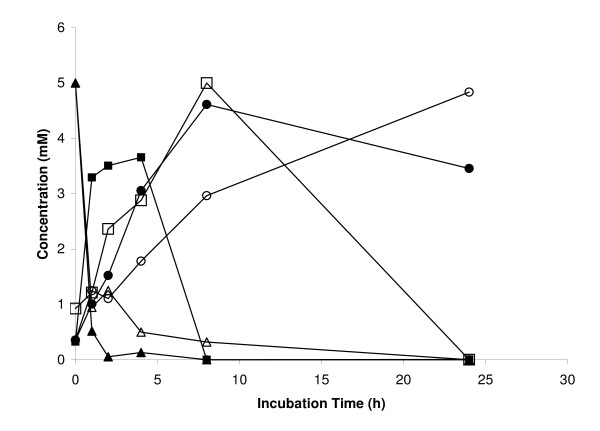
**Nitrate reduction in resting cell assays with the wild type (closed symbols) and the ETRA7-1 (open symbols) mutant strains**. Nitrate - triangles, nitrite - squares and ammonium - circles. Nitrate measurements in killed controls did not change, while nitrite and ammonium were not detected (data not shown).

Anaerobic cultures of the mutant and the wild type strain were analyzed for the reduction of different electron acceptors with lactate as the electron donor. No growth of the EtrA7-1 mutant was observed with fumarate as electron acceptor whereas the wild type strain reached an OD_600 _of 0.053 ± 0.005. Limited growth (approximately 50% lower OD_600 _compared with the wild type cultures) was observed in mutant cultures amended with trimethylamine N-oxide (TMAO) or thiosulfate (data not shown). No OD increases with the mutant and the wild type were measured with DMSO provided as electron acceptor at 2 and 10 mM; however, HPLC analyses of cultures with 2 mM DMSO revealed that DMSO was completely consumed in wild type cultures, whereas no DMSO consumption was evident in the mutant cultures (Figure 3). No changes in DMSO concentrations were observed in cultures with 10 mM DMSO. No significant differences in Fe(III), Mn(IV) and sulfite reduction rates were observed between the wild type and the EtrA7-1 deletion mutant (Figure 3). Anaerobic cultures of the mutant and the wild type strains grown with pyruvate instead of lactate as electron donor showed similar results, i.e., the mutant showed limited or no growth with nitrate, fumarate and DMSO provided as electron acceptors compared to the wild type (Figure 4). Similar to the lactate-amended cultures, the rates of nitrate, fumarate and DMSO reduction in wild type cultures exceeded those measured in cultures of the mutant strain (Table 1). Resting cell assays corroborated these findings and nitrate reduction and ammonium production occurred at higher rates in assays with wild type cells. Complete stoichiometric conversion to ammonium also occurred in the assays with mutant cells, although lower rates and a 3-fold longer incubation were required for complete reduction (i.e., 24 h for the EtrA7-1 versus 8 h for the wild type) (Figure 5).

**Table 1:** Comparison of reduction rates of several electron acceptors with pyruvate as electron donor by *S. oneidensis *MR-1 wild type strain and *etrA *knockout strain EtrA7-1.

Electron acceptor	**Wild type (μM min**^**-1**^**)**	**ETRA7-1 (μM min**^**-1**^**)**
Nitrate	1.2 ± 0.1	0.3 ± 0.01
Fumarate	6.4 ± 0.6	3.8 ± 0.2
DMSO	0.8 ± 0.2	0.4 ± 0.1

Data are means ± the standard deviation from three independent cultures.

### Effects of *etrA *deletion on transcription

The global transcriptome profile of mutant strain EtrA7-1 grown anaerobically with nitrate as the sole electron acceptor was compared to that of the wild type under the same growth conditions. A complete list of all the genes differentially expressed two-fold or higher is provided as supplemental information (Additional file 1). Out of 612 differentially transcribed genes in the EtrA7-1 mutant relative to the wild type, 289 were up-regulated and 323 were down-regulated. The differentially transcribed genes were classified in 19 functional "TIGR Role" categories (Additional file 2) based on the MR-1 genome annotation (GenBank accession number AE014299) [22]. Genes with unknown functions represented the largest category of up-regulated (14.8%) and the second most common category of down-regulated genes (17.3%). Genes associated with energy metabolism were the largest category (17.6%) of down-regulated genes (Additional file 2). Among the up-regulated genes, the "Protein synthesis" category ranked second (12.5%) and the "Other categories" ranked third (11.4%). This latter category included phage-, transposase- and plasmid-related genes. The "Energy metabolism" category represented 9.7%, ranking fourth.

### Identification of putative EtrA binding sites

The promoters of the differentially expressed genes were examined for putative EtrA binding sites in order to identify those genes that were likely directly regulated by EtrA from the many genes whose expression changes were most likely due to secondary effects. For example, the up-regulation of phage-related genes is likely a response to stress, and not a direct result of the *etrA *deletion. Putative EtrA binding sites were identified for those genes that showed at least 2.5 fold change in the EtrA7-1 mutant relative to the wild type by extracting the upstream intergenic sequences and applying the Gibbs centroid sampler to identify a putative binding site motif. A motif was identified (Additional file 3) that displays similarities to the *E. coli *Fnr and Crp binding sites motifs; this motif was present upstream of 44 operons that encode a total of 78 genes. The largest proportion of these genes is in the "Energy metabolism" category (Table 2 and 3, Additional file 2). Binding sites were detected upstream of an additional 28 operons when the detected motif (Additional file 3) was used to scan the upstream intergenic regions of all genes listed in Additional file 1.

**Table 2:** Genes induced in the "Energy Metabolism" category in anaerobic cultures of EtrA7-1 relative to the wild type (reference strain).

Gene ID	Gene name	**Relative expression**^**a**^	**Predicted EtrA binding sites**^**c**^	COG Annotation
SO0162	*pckA*	2.21 (± 0.48)^b^	TGTGAGCTGGATCATT	phosphoenolpyruvate carboxykinase (ATP)
SO0747	*fpr*	2.17 (± 1.01)		ferredoxin--NADP reductase
SO1103	*nqrA-2*	2.25 (± 0.54)	TCTGCGCTAGCTCAAT CGTGATTGCGATCGCA	NADH:ubiquinone oxidoreductase, Na translocating, alpha subunit
SO1104	*nqrB-2*	2.70 (± 1.01)	↓	NADH:ubiquinone oxidoreductase, Na translocating, hydrophobic membrane protein NqrB
SO1105	*nqrC-2*	3.15 (± .080)	↓	NADH:ubiquinone oxidoreductase, Na translocating, gamma subunit
SO1106	*nqrD-2*	4.65 (± 2.07)	↓	NADH:ubiquinone oxidoreductase, Na translocating, hydrophobic membrane protein NqrD
SO1107	*nqrE-2*	3.63 (± 1.61)	↓	NADH:ubiquinone oxidoreductase, Na translocating, hydrophobic membrane protein NqrE
SO1108	*nqrF-2*	4.21 (± 2.05)	↓	NADH:ubiquinone oxidoreductase, Na translocating, beta subunit
SO1891	*scoB*	3.77 (± 1.80)		Acetyl-CoA:acetoacetate CoA transferase, alpha subunit AtoA
SO1892	*scoA*	3.21 (± 2.14)		acetate CoA-transferase, beta subunit AtoD
SO1927	*sdhC*	2.47 (± 1.26)		succinate dehydrogenase, cytochrome b556 subunit
SO1930	*sucA*	3.02 (± 1.22)		2-oxoglutarate dehydrogenase, E1 component
SO1931	*sucB*	3.60 (± 1.58)		2-oxoglutarate dehydrogenase, E2 component, dihydrolipoamide succinyltransferase
SO1932	*sucC*	3.29 (± 0.98)		succinyl-CoA synthase, beta subunit
SO1933	*sucD*	3.28 (± 1.24)		succinyl-CoA synthase, alpha subunit
SO2361	*ccoP*	2.30 (± 0.92)	↑	cytochrome c oxidase, cbb3-type, subunit III
SO2362	*ccoQ*	3.44 (± 1.16)	↑	cytochrome c oxidase, cbb3-type, CcoQ subunit
SO2364	*ccoN*	2.76 (± 1.07)	CTTGAGCCATGTCAAA GTTGATCTAGATCAAT	cytochrome c oxidase, cbb3-type, subunit I
SO4509	*fdhA-1*	2.33 (± 0.56)		formate dehydrogenase, alpha subunit
SO4510	*fdhB-1*	4.03 (± 1.57)		formate dehydrogenase, iron-sulfur subunit
SO4511	*fdhC-1*	2.53 (± 0.31)		formate dehydrogenase, C subunit, putative

^a ^The relative expression is presented as the ratio of the dye intensity of the anaerobic cultures with 2 mM KNO_3 _of EtrA7-1 to that of MR-1 (reference).

^b ^The standard deviation was calculated from six data points, which included three independent biological samples and two technical samples for each biological sample.

^c ^The arrows indicate that the gene is regulated by the binding site that follows. The direction of the arrow indicates the location of the gene. An arrow pointing down indicates the gene or operon is in the plus or sense strand and the arrow pointing up indicates the gene or operon is in the minus or anti-sense strand.

**Table 3:** Genes repressed in the "Energy metabolism" category in anaerobic cultures of EtrA7-1 grown on lactate and nitrate relative to the wild type (reference strain).

Gene ID	Gene name	**Relative expression**^**a**^	**Predicted EtrA binding sites**^**c**^	COG Annotation
SO0274	*ppc*	0.48 (± 0.19)		phosphoenolpyruvate carboxylase
SO0398	*frdA*	0.30 (±0.16)^b^		fumarate reductase flavoprotein subunit
SO0399	*frdB*	0.39 (± 0.06)		fumarate reductase iron-sulfur protein
SO0845	*napB*	0.15 (± 0.04)		cytochrome c-type protein NapB
SO0846	*napH*	0.18 (± 0.11)		iron-sulfur cluster-binding protein napH
SO0847	*napG*	0.14 (± 0.07)		iron-sulfur cluster-binding protein NapG
SO0848	*napA*	0.18 (± 0.13)	↑	periplasmic nitrate reductase
SO0849	*napD*	0.30 (± 0.04)	GTCGATCGGGATCAAA CGTGATCTAACTCTCA	napD protein
SO0903	*nqrB-1*	0.34 (± 0.15)	TTTGCTGTAAAGCAAA TGTGCATGGAATCGCC	NADH:ubiquinone oxidoreductase, Na translocating, hydrophobic membrane protein NqrB
SO0904	*nqrC-1*	0.28 (± 0.09)	↓	NADH:ubiquinone oxidoreductase, Na translocating, gamma subunit
SO0905	*nqrD-1*	0.27 (± 0.14)	↓	NADH:ubiquinone oxidoreductase, Na translocating, hydrophobic membrane protein NqrD
SO0906	*nqrE-1*	0.23 (± 0.07)	↓	NADH:ubiquinone oxidoreductase, Na translocating, hydrophobic membrane protein NqrE
SO0907	*nqrF-1*	0.23 (± 0.08)		NADH:ubiquinone oxidoreductase, Na translocating, beta subunit
SO0970	*fccA*	0.31 (±0.17)		Periplasmic fumarate reductase, FccA
SO1018	*nuoE*	0.44 (± 0.17)		NADH dehydrogenase I, E subunit
SO1019	*nuoCD*	0.35 (± 0.13)		NADH dehydrogenase I, C/D subunits
SO1020	*nuoB*	0.40 (± 0.10)		NADH dehydrogenase I, B subunit
SO1363	*hcp*	0.13 (± 0.08)		prismane protein
SO1364	*hcr*	0.12 (± 0.07)		iron-sulfur cluster-binding protein
SO1429	*dmsA-1*	0.43 (± 0.09)	TGTGATACAATTCAAA	anaerobic dimethyl sulfoxide reductase, A subunit
SO1430	*dmsB-1*	0.29 (± 0.04)	↓	anaerobic dimethyl sulfoxide reductase, B subunit
SO1490	*adhB*	0.28 (± 0.12)	TGTGATCTAGATCGGT TTGGAACTAGATAACT	alcohol dehydrogenase II
SO1776	*mtrB*	0.22 (± 0.04)		outer membrane protein precursor MtrB
SO1777	*mtrA*	0.25 (± 0.06)		decaheme cytochrome c MtrA
SO1778	*mtrC*	0.30 (± 0.09)		decaheme cytochrome c MtrC
SO1779	*omcA*	0.30 (± 0.05)	GTGGAATTAGATCCCA TGTGATTGAGATCTGA TTTGAGGTAGATAACA	decaheme cytochrome c
SO2097	*hyaC*	0.07 (± 0.04)		quinone-reactive Ni/Fe hydrogenase, cytochrome b subunit
SO2098	*hyaB*	0.11 (± 0.10)		quinone-reactive Ni/Fe hydrogenase, large subunit
SO2099	*hyaA*	0.07 (± 0.11)		quinone-reactive Ni/Fe hydrogenase, small subunit precursor
SO2136	*adhE*	0.40 (± 0.10)		aldehyde-alcohol dehydrogenase
SO2912	*pflB*	0.18 (± 0.11)	TTTGAGCTGAAACAAA	formate acetyltransferase
SO2913	*pflA*	0.20 (± 0.13)		pyruvate formate-lyase 1 activating enzyme
SO2915	*ackA*	0.23 (±0.16)		acetate kinase
SO2916	*pta*	0.23 (± 0.14)		phosphate acetyltransferase
SO3144	*etfA*	0.36 (± 0.13)		electron transfer flavoprotein, alpha subunit
SO3285	*cydB*	0.21 (± 0.06)	↑	cytochrome d ubiquinol oxidase, subunit II
SO3286	*cydA*	0.22 (± 0.10)	TTTGATTCAAATCAAT	cytochrome d ubiquinol oxidase, subunit I
SO3980	*nrfA*	0.18 (± 0.06)	TTTGCGCTAGATCAAA	cytochrome c552 nitrite reductase
SO4513	*fdhA-2*	0.06 (± 0.02)	ACTGTTCTAGATCAAA	formate dehydrogenase, alpha subunit
SO4515	*fdhC-2*	0.07 (± 0.01)		formate dehydrogenase, C subunit, putative
SO4591	*cymA*	0.39 (± 0.27)		tetraheme cytochrome c

^a ^The relative expression is presented as the ratio of the dye intensity of the anaerobic cultures with 2 mM KNO_3 _of EtrA7-1 to that of MR-1 (reference).

^b^The standard deviation was calculated from six data points, which included three independent biological samples and two technical samples for each biological sample.

^c ^The arrows indicate that the gene is regulated by the binding site that follows. The direction of the arrow indicates the location of the gene. An arrow pointing down indicates the gene or operon is in the plus or sense strand and the arrow pointing up indicates the gene or operon is in the minus or anti-sense strand.

### Regulatory role of EtrA in energy metabolism

Since the "Energy metabolism" category contained the largest group of genes responsive to EtrA, these genes were analyzed in more detail. Up-regulated genes (Table 2) in this group included genes encoding a cytochrome *c *oxidase (*ccoPQN *[SO2361-2362, SO2364]), proteins involved in gluconeogenesis such as PckA (SO0162), and *nqrABCDEF*-2 genes (SO1103-1108) encoding NADH:ubiquinone oxidoreductases. From this group, only the *nqr *gene clusters had a putative EtrA binding site.

While the *nqr-*2 gene cluster was up-regulated in the *etrA *knockout mutant, the *nqr-*1 gene cluster (SO0903-0907) was down-regulated. Nqr is a Na^+ ^pump that during respiration generates a sodium motive force to mediate solute transport, flagellar motility and ATP synthesis [23]. Both *nqr *gene clusters had putative EtrA binding sites. The microarray data indicated that EtrA affects the transcription pattern of these genes differently. Similarly, the *etrA *deletion had a distinct effect on the expression of the *fdh *gene clusters encoding a formate dehydrogenase. The *fdh*-1 genes (SO4508-4511) were up-regulated whereas the *fdh*-2 gene cluster (SO4512-4515) was down-regulated. An EtrA binding site was only identified for the *fdh*-2 cluster and not for the *fdh-1 *cluster, indicating EtrA affects both clusters differently.

Other up-regulated genes in the "Energy metabolism" category included the succinate dehydrogenase gene *sdhC *(SO1927), the succinyl-CoA synthase operon *sucABCD *(SO1930-1933), the butyryl-CoA:acetate CoA-transferase and the acetyl CoA-synthase genes (SO1891-1892).

Down-regulated genes in the "Energy metabolism" category (Table 3), which were predicted for direct regulation by EtrA, included genes involved in anaerobic metabolism, especially in nitrate respiration like the *napBHGAD *operon (SO0845-0849, strongly repressed with all subunit genes except *napD *with ratios ≤ 0.18), and the *nrfA *(SO3980) genes. *cymA *(SO4591; ratio 0.39), the prismane protein *hcp *gene (SO1363), and neighboring protein *hcr *gene (SO1364), both of which were strongly repressed (ratios ≤ 0.13) and have been associated with the nitrate reduction pathway [24-27], did not show evidence of EtrA binding sites. Also indirectly down-regulated were the fumarate reductase genes *frdAB *(SO0398-0399) and *fccA *(SO0970), the *ackA *and the *pta *(SO2915-16) genes involved in acetate production and the *ppc *(SO0274) gene encoding an acetate phosphoenol pyruvate carboxylase. The *hyaCBA *(SO2097-2099) genes encoding a quinone-reactive Ni/Fe hydrogenase were highly indirectly repressed (ratio ≤ 0.11). Among the genes identified as directly down-regulated are all the genes in the operon that encodes the anaerobic DMSO reductase (*dmsAB*) (SO1428-32), the *cydAB *genes (SO3285-3286) encoding a cytochrome *d *oxidase complex, as well as genes involved in metabolism of organic compounds such as the *pflAB *(SO2912-2913).

Other down-regulated genes grouped in different categories included genes encoding ABC transporters (*cydCD *[SO3779-3780], SO4446-4448), TonB-dependent receptors (*nosA *[SO0630]), and L-lactate permease (*lldP *[SO0827]) and a putative lactate permease (SO1522). The only gene directly down-regulated from this later group is *lldP *(SO0827), for which an EtrA binding site was predicted (Table 3). As expected, the cDNA for *etrA*, shows no significant hybridization signal in EtrA7-1 mutant (ratio 0.05).

### Stress response caused by the *etrA *deletion

We detected induction of genes from various categories, which have been associated with stress response i.e., starvation, phage infection and oxidative stress, possibly due to accumulation of nitrogen oxide reactive species. Up-regulated genes (Additional file 1) were dominated by genes grouped in "Other categories". The majority of up-regulated genes were phage-related. For example, 25 genes of the LambdaSo phage (SO2940-2974), a gene encoding a viral capsid protein of the MuSo1 phage (SO0675), and genes of MuSo2 phage (SO2684-2685, SO2687, SO2702) were up-regulated. In contrast, the gene encoding the LambdaSo phage transcriptional regulator of the Cro/CI family (SO2990) was down-regulated (ratio 0.43). Transcriptional changes of most of these genes are likely indirect effects due to the deletion of the *etrA *gene and only for the LambdaSo phage genes S02957-2962 was an EtrA binding site predicted.

The category "Transport and binding proteins" contains a large number of genes associated with stress response. Up-regulated genes in this group contain genes encoding heavy metal efflux pumps (SO0520, SO4596-4598, SOA0153) and genes for phosphate transport (SO1560, SO1723-1724 including *pstB-1 *[SO1725], *pstB-2 *[SO4289] and *pstA *[SO4290]). There was up-regulation of *phoBR *(SO1558-59) and *phoU *(SO1726) genes, which regulate the phosphate transporters genes during phosphate starvation [28-32].

Up-regulated genes in response to stress conditions i.e., starvation, phage infection, oxidative stress, include a stringent starvation protein encoded by the *sspAB *genes (SO0611-0612)[33], and a phage shock protein operon *pspABC *(SO1807-1809)[34]. Other up-regulated stress-related genes were the RNA polymerase sigma-70 factor *rpoD *(SO1284)[32,35], a GTP-binding protein that regulates the TCA cycle and responds to starvation (*era *[SO1349])[36], and a DNA repair protein (*recO *[SO1350])[37].

## Discussion

The results of this study demonstrate that EtrA positively regulates dissimilatory nitrate, fumarate and DMSO reduction pathways in *S. oneidensis *MR-1. The generation of *etrA *knockout mutant EtrA7-1 in the wild type strain MR-1 background eliminated any possible secondary effects on the phenotype, such as the electron transfer perturbation suspected with the rifampicin resistant DSP10 strain [6]. Similar to other *etrA *mutants of strain MR-1, EtrA7-1 retained its ability to reduce nitrate [6,7,16]; however, our results show that the anaerobic growth of the mutant was significantly impaired compared to the wild type when nitrate was the only electron acceptor. Likewise, the *etrA *deletion mutant lost its ability to reduce fumarate and DMSO with both lactate and pyruvate as electron donor. Regulation of DMSO reduction by EtrA in strain MR-1 was suggested previously [6] however this study provides physiological evidence that confirm its role. The ability of the EtrA7-1 mutant to reduce TMAO and thiosulfate also decreased; however the reduction of Fe(III) citrate, Mn(IV) and sulfite was not affected by the deletion. No differences in growth performance between the wild type and the mutant were observed under aerobic conditions (data not shown).

The transcriptome analysis provides a genome-wide expression profile of *S. oneidensis *MR-1 instead of the partial genome array that was previously evaluated (691 ORFs [6] vs 4,648 genes in this study). We observed in 612 (13%) differentially expressed genes represented though some are likely due to differences in growth rate between the mutant strain and the wild type strain. Nonetheless, the expression patterns of genes are consistent with the physiological data and with the transcription data reported for Fnr in *E. coli *[11,12,20] and with the more limited data by Beliaev et al. [6]. Genes involved in nitrate reduction (*napDAHGB, nrfA*, and *hcp*) were significantly down-regulated by the *etrA *deletion as well as those encoding the fumarate reduction (*frdAB, fccA*) and all the genes encoding for the DMSO reductases (*dmsAB*). All of these genes have been considered candidates for EtrA regulation in previous studies; however, results were not conclusive [5-7,16]. Sequence analysis of the regulatory regions of the differentially expressed genes, indicated possible EtrA recognition sites for most genes in the "Energy metabolism" category, some of which i.e. *napDAHGB, nrfA, frdAB and dmsAB*, confirms previous results [6] and further suggests that regulation of these genes is via direct interaction of EtrA with their promoters. Putative recognition sites for EtrA were also identified for the two *nqr *gene clusters, which had not been identified previously. Also, the regulatory regions for *fdh *gene clusters were evaluated and an EtrA binding site was recognized for only *fdhA*-1. The *fdh*-2 cluster does not possess an EtrA binding site, suggesting a different regulatory system.

Our data indicate that EtrA is a global regulator acting in cooperation with other regulatory proteins to control anaerobic metabolic processes in strain MR-1 [6,7,16], therefore, the expression of these genes cannot be expected to be under an "all or none" regulatory mechanism. Rather, these global regulators respond to multiple stimuli (e.g., oxygen levels, substrates) and fine-tune regulation via transcriptional control and interactions between regulatory proteins. Studies in *S. oneidensis *and in other *Shewanella *species that indicate the combined action of transcriptional regulators for the anaerobic metabolism in this organism [4,17-19]. For example, recent studies showed that CRP, EtrA and the product of the *cya *genes act as expression regulators of several anaerobic respiratory systems, including nitrate reduction in *S. oneidensis *MR-1 and *Shewanella *sp. strain ANA-3 [4,17-19]. In *E. coli*, Fnr and NarP positively regulate the *nap *and *nrf *genes [12,20,38,39]. MR-1 possesses the genes for a homolog of the two-component regulatory system in *E. coli *NarQ/NarP (SO3981-3982). The presence of alternate regulators that partially fulfill the function of EtrA can explain why nitrate reduction even though impaired, still occurred in the EtrA7-1 knockout mutant.

Down-regulation of genes for lactate transport was also observed. Since lactate was the source of reducing equivalents and carbon, a lack of electron donor and carbon may have contributed to the impaired growth of the EtrA7-1 mutant. Induction of transport proteins for carbon sources and electron acceptors has also been credited to Fnr in *E. coli *[12,20], and a putative EtrA binding site was predicted for the gene encoding a lactate permease (SO0827) in MR-1.

Impaired growth of EtrA7-1 could also be due to stress factors caused or enhanced by the deletion (e.g. accumulation of nitrogen oxide reactive species and starvation). The expression of phage-related genes induced in response to irradiation in strain MR-1 has been reported [40]. Up-regulation of the genes involved in activation of the strain MR-1 prophages LambdaSo, MuSo1 and MuSo2 in the EtrA7-1 mutant was observed, suggesting phage activity. Induction of bacterial genes (e.g., *nusAG*) required to stabilize the Lambda protein antitermination complex in *E. coli *was also shown [41,42]. Conversely, there is repression of the LambdaSo transcription regulator Cro/CI family, which represses the transcription of the Lambda genes in *E. coli *[43]. Also, the induction of genes associated with starvation, i.e., a condition that could activate the lytic cycle of prophages [43], was confirmed in the expression analysis.

## Conclusion

The involvement of several regulatory controls has complicated the interpretation of gene expression patterns and functions in *Shewanella *spp. Results from the above *etrA *deletion mutant studies suggest a global regulatory role for EtrA, but one which works in conjunction with other regulators to fine-tune the expression of key genes in anaerobic metabolic pathways in *S. oneidensis *strain MR-1. Besides confirming and clarifying previous reports on Fnr regulation, we also provide experimental evidence for a positive regulatory role of EtrA in the DMSO reduction pathway of strain MR-1. Furthermore, our whole-genome transcriptional profile shows the effects of EtrA on the expression of genes not previously evaluated (e.g. *nqr*, *fdh*-1, phage- and stress-related genes), and differences in the expression pattern of genes previously analyzed (e.g. *cydAB *and *sdhC*)[6,12]. These observations are consistent with results obtained by Gralnick et al. [4] suggesting a distinctive regulatory system, although very similar to Fnr in *E. coli*. A stringent sequence analysis of the regulatory region of the genes affected by the mutation suggest direct interaction of EtrA to those in the "Energy metabolism" category, while stress- and phage-related genes are up-regulated indirectly as a consequence of a secondary perturbation. This and previous work taken together suggest that this regulator is more properly termed Fnr.

## Methods

### Bacterial strains and culture conditions

The bacterial strains, plasmids, primers and, probes used in this study are described in Table 4. *S. oneidensis *strain MR-1 and its mutant strains were grown in HEPES medium as described [44]. The medium was supplemented with 20 mM lactate and KNO_3 _was added as electron acceptor in concentrations specified below. Oxygen was removed from the medium by boiling and purging with helium [45]. Cultures of *E. coli *strain β2155 (auxotroph of diaminopimelic acid [DAP]) were grown in Luria-Bertani (LB) medium supplemented with 100 μg/ml of DAP at 37°C. *S. oneidensis *strain MR-1 was cultivated in aerobic LB medium at 30°C during the mutagenesis process. Antibiotics used for the selection of MR-1 transformants were added in the following concentrations: 25 μg/ml of kanamycin, 7.5 μg/ml of gentamycin, and 10 μg/ml of tetracycline. Vessels that received no inoculum or no KNO_3 _served as negative controls.

**Table 4:** Bacterial strains, plasmids, primers and oligonucleotides used in this study.

Strain/plasmid/primer/Probe	**Relevant genotype or sequence**^**a,b**^	Source
*Escherichia coli *K-12		
β2155	DAP auxotroph	[61]
*Shewanella oneidensis*		
MR-1 (ATCC 700550^T^)	wild type	[62]
EtrA7-1	As MR-1 but Δ*etrA*::*loxP*	This study
EtrA7-1 complement	EtrA7-1 complemented with the *etrA *gene (SO2356) cloned into	This study
	pCM62	
EtrA7-1 with pCM62	EtrA7-1 harboring the pCM62 as a negative control for complementation	This study
Plasmids		
pCM62	Tc^r^; *traJ' trfA oriT oriV*	[63]
pCM157	Tc^r^; *trfA oriT oriV *ColE1 *ori; lacZ*p-*cre*	[46]
pCM184	Ap^r ^Tc^r^; *loxP-*kan-*loxP*	[46]
pKNOCK-Gm	Gm^r^; *ori*_R6K _*oriT*_RP4_	[64]
pCCG02	As pKNOCK-Gm but *etrA'*-*loxP-*kan-*loxP-'etrA*	This study
pCCG03	As pCM62 but *lacZ*p-*etrA*	This study
Primers^c^		
etrAN Fwd	GCCGCGGTCATGTCGGTTCTCAAGT	
etrAN Rev	CGAGCTCCGACAGCTATCTGTTAGTCT	
etrAC Fwd	CGAATTCAAATCACCGCTTTTAACTTG	
etrAC Rev	GCATATGCCAGATAAATCACACCTTTT	
etrAScreenout	AATTCTTCAGGCATTTGACTCG	
Fwd		
etrAScreenout	GGCCGTATCTTGAGTTATACCC	
Rev		
etrAcomp Fwd	GGATCCAGGTGTGATTTATCTGGCG	
etrAcomp Rev	GAATTCCCGACATGACAATAGAGCAGA	
23SRT Fwd	TAGCGAAATTCCTTGTCGGG	
23SRT Rev	GAGACAGCGTGGCCATCATT	
23Stemp Rev	GTATCAGTTAGCTCAACGCCTC	
napART Fwd	AGAAAGCCCTGTTAACCGTGG	
napART Rev	TCATCCGCAGCAATGGTGT	
napAtemp Rev	GATCGAAGCTACGGTTCTCG	
nrfART Fwd	GCCACATGTATGCCGTGACT	
nrfART Rev	TTTACAGCTCCAGCAAGCCA	
nrfAtemp Rev	ACGTTTCATACTCGGGATGC	
Probes		
23SRTProbe	AGTTCCGACCTGCACGAATGGCG	
napARTProbe	CTGTATTAAAGGTTACTTCCTGTCGAAAATCATGTACGG	
nrfARTProbe	CGTAATACCTTGCGTACTGGCGCGC	

^a ^The sequence for the primers is written from the 5'end to the 3'end.

^b ^Primers were designed using putative gene sequences of *S. oneidensis *MR-1.

^c ^For primer sequences, the restriction sites incorporated are underlined. CATATG, NdeI; GAATTC, EcoRI; GAGCTC, SacI; CCGCGG, SacII.

### Construction of a chromosomal Δ*etrA*::*loxP *allele

PCR primers were designed with Vector NTI^® ^software (InforMax, Inc., Frederick, MD) and synthesized at Integrated DNA Technologies (http://www.idtdna.com).

A deletion mutant of the *etrA *gene (SO2356) was constructed by allelic replacement as described [44]. Primers *etrA*N Rev (*Sac*I) and *etrA*N Fwd *(Sac*II) generated a 520 bp fragment containing about two-thirds of the upstream SO2357 gene, the SO2357-*etrA *intergenic region, and the first four base pairs of the 753-bp *etrA *gene (Table 4). Primers *etrA*C Rev (*Nde*II) and *etrA*C Fwd (*Eco*RI) generated a 526 bp fragment containing the last five base pairs of the *etrA *gene, the *etrA*-SO2355 intergenic region and about half of the downstream SO2355 gene (Table 4). The resulting 'SO2357*-etrA'-loxP-kan-loxP-'etrA-*SO2355*' *assembly was cloned into the conditionally-replicating plasmid pKNOCK-Gm (Table 4), which encodes resistance to gentamycin, to generate plasmid pCCG02. *S. oneidensis *MR-1 Km^r ^Gm^s ^colonies were screened by PCR using primers *etrA*Screenout Fwd and *etrA*Screenout Rev (Table 4) to identify recombinants in which the *etrA *gene was replaced by the loxP-kan-loxP cassette.

The *kan *gene was subsequently removed from the mutant MR-1 genome by Cre recombination [44,46]. PCR and DNA sequence analyses confirmed the presence of the chromosomal Δ*etrA*::*loxP *allele. DNA sequencing was performed at the Genomics Technical Support Facility at Michigan State University.

### Δ*etrA*::*loxP *mutant complementation

Plasmid pCM62 (Table 4) was used as the vector for the expression of the *etrA *gene in a Δ*etrA*::*loxP *mutant (strain EtrA7-1). The *etrA *gene (SO2356) was PCR amplified from *S. oneidensis *MR-1 genomic DNA using the etrAcomp Fwd (*Bam*HI) and etrAcomp Rev (*Eco*RI)(Table 4). The amplicon was double digested with *Bam*HI and *Eco*RI and ligated to the multiple cloning site in pCM62. This construct (pCCG03) was transformed into EtrA7-1 by conjugation from *E. coli *β2155. Ligation, electroporation into *E. coli *β2155, and conjugation in strain EtrA7-1 were performed as described [44]. Plasmid pCM62 was also transformed into EtrA7-1 via conjugation from *E. coli *β2155 and used as a control for any plasmid effects. Transformants were selected by streaking on LB plates with tetracycline. EtrA7-1 Tc^r ^colonies were diagnosed by PCR using the etrAcomp primers (Table 4) and subsequently sequenced to verify the deletion of the *etrA *gene.

### Phenotypic characterization of the Δ*etrA*::*loxP *mutant

Cultures of the wild type, EtrA7-1, EtrA7-1 complement and EtrA7-1 harboring pCM62 were grown anaerobically with 3 mM KNO_3 _in HEPES medium. Growth was monitored periodically by OD measurements at 600 nm. Samples (2 mL) were periodically withdrawn for analysis of nitrate, nitrite and ammonium concentrations as described [44,47].

Cultures of the wild type and EtrA7-1 were also cultivated anaerobically with ferric citrate (10 mM), fumarate (10 mM), disodium thiosulfate (10 mM), trimethylamine *N-*oxide (TMAO; 10 mM), manganese dioxide (1 mM, nominal concentration), dimethyl sulfoxide (DMSO; 2 and 10 mM) and disodium sulfite (1 mM), as electron acceptors. The ferric citrate and the manganese dioxide were prepared as described [48]. Evidence of growth via reduction of TMAO, thiosulfate and fumarate was determined by OD_600 _measurements. Fe(III) reduction was determined by the ferrozine assay following HCl extraction [49,50]. Mn(IV) reduction was assayed colorimetrically [48]. Cultures supplied with DMSO as the terminal electron acceptor were analyzed by high-performance liquid chromatography (HPLC) for lactate consumption and acetate formation [51]. Sulfite consumption was measured using a DX-100 ion chromatograph (Dionex Corp., Sunnyvale, CA) equipped with an IonPac AS14A Column.

To determine the effects of lactate on the reduction of DMSO, nitrate and fumarate, cultures of the wild type and the EtrA7-1 mutant strain were grown anaerobically with 20 mM sodium pyruvate as the electron donor and dimethyl sulfoxide (DMSO; 1 mM), fumarate (10 mM) or nitrate (2 mM) as electron acceptors. DMSO and fumarate reduction were monitored as mentioned above. Nitrate reduction was measured using a Dionex ICS-3000 ion chromatograph (Dionex Corp., Sunnyvale, CA) equipped with an IonPac AS14 Column.

### Resting cell assays

Cells of the wild type strain MR-1 and the EtrA7-1 mutant were grown aerobically in HEPES medium with 20 mM lactate as the electron donor to OD_600 _= 0.2. Cells were harvested by centrifuging for 10 min at 3,000 *g*, washed twice with 50 mM saline phosphate buffer (pH 7.0) [52,53], and resuspended in the same buffer to an OD_600 _= 1.0 under anoxic conditions. The cells were incubated with 5 mM KNO_3_, and after 0, 1, 2, 4, 8 and 24 h were removed by centrifugation and three 1-mL replicate samples of the supernatant were assayed to determine nitrate and nitrite reduction rates. Assays with autoclaved wild type cells served as negative controls. Nitrate, nitrite and ammonium concentrations were determined as described [44].

### Total RNA preparations

Total RNA was extracted from triplicate cultures of strains MR-1 and EtrA7-1 grown with 2 mM nitrate as the sole electron acceptor. The RNA was extracted with RNAwiz Solution following the instructions of the manufacturer (Ambion, Inc., Austin, TX). RNA samples were treated with RNase-free DNaseI (Roche Pharmaceuticals, Basel, Switzerland) and purified by phenol:chloroform (1:1) and chloroform extractions [54], and stored in ethanol at -80°C until use. Quality of the RNA was verified using the RNA 6000 Pico LabChip kit and the 2100 Bioanalyzer (Agilent Technologies, Inc., Santa Clara, CA).

### Global expression analyses

A *S. oneidensis *strain MR-1 whole genome microarrays [55] were provided by Liyou Wu and Jizhong Zhou (Oak Ridge National Laboratory, Oak Ridge, TN). cDNA preparation and labeling were performed as described [56] using a 2:3 ratio of 5-(3-aminoallyl)-dUTP and dTTP. Hybridization and post-hybridization washes were done as described [57]. Three biological replicates per treatment were used for the hybridization of six microarray slides including technical duplicates (dye-swap). Data analysis was performed using the GeneSpring 6.0 software (Silicon Genetics, Redwood City, CA). The data were normalized per chip and per gene (Lowess Normalization) and the spots with less than 55% pixel intensity above background plus two standard deviations were eliminated from the analyses [58]. The data were filtered using the Benjamini and Hochberg false discovery rate with 95% confidence and only those genes with a > 2-fold change in expression were considered significant.

### Microarray data accession number

The raw microarray intensity data has been deposited in the GenBank Gene Expression Omnibus (GEO) database under the accession number GSE26935.

### Identification of putative EtrA binding sites

Regulatory motifs were predicted in the intergenic regions of differentially expressed genes using the Gibbs centroid sampler [59]. Intergenic regions were extracted, based on the *S. oneidensis *MR-1 genome annotation, that were at least 50 bp in length and upstream of differentially expressed genes or operons whose change in expression (average ± one standard deviation) was at least 2.5-fold. A total of 170 genes met these requirements: 55 had expression levels minus one standard deviation of ≥ 2.5 (up-regulated genes) and 115 had expression levels plus one standard deviation of ≤ 0.4 (down-regulated genes). For these genes, 118 upstream intergenic regions were extracted for analysis, after accounting for multiple genes within an operon. The parameters for the Gibbs centroid sampler used on these sequences were the following: up to two motif models were allowed, where each model was specified to be palindromic and 16-24 bases long, a maximum of three sites per intergenic was allowed, a position-specific background model [60] was employed, and centroid sampling was performed with 1000 burn-in iterations, 5000 sampling iterations and 10 random seeds. The results from four independent runs were compared, and the subset of 47 intergenic regions extracted that contained a predicted regulatory motif in at least one of those runs. These 47 intergenic sequences were analyzed with the Gibbs centroid sampler, using the same parameters as above, except that only one motif model was specified. Additional binding sites were detected using dscan (http://ccmbweb.ccv.brown.edu/cgi-bin/dscan.pl) to search the set of promoters for all the genes that exhibited ≥ 2-fold change in expression (Additional file 1). This set included a total of 424 intergenic regions.

## Authors' contributions

All authors contributed in the organization and design of experiments as well as data interpretation and manuscript preparation. CCG, FEL, and JMT wrote the paper. CCG designed and carried out the majority of the experimental work including mutant construction, cDNA microarray experiments and analysis, and growth studies. AEM, MFR and LAM contributed in experimental design and cDNA microarray data analysis and interpretation. JLMR performed resting cell assays. JAG replicated DMSO physiological experiments. LAM performed EtrA binding site identification. MFR provided updated genome sequence annotation. FEL provided laboratory equipment, materials, and funding and supervision for the phenotypic characterization work. JMT supervised experimental work. All authors read and approved the final version of the manuscript.

## Supplementary Material

Additional file 1 **Supplemental Table SI1. Genes differentially expressed in anaerobic cultures of MR-1 and Etra7-1 at different concentrations of KNO3**. Complete list of genes differentially expressed including relative expression, standard deviation, "TIGR role" and predicted EtrA binding sites. Click here for file

Additional file 2 **Figure SI1. Distribution of differentially expressed genes (> 2-fold change) grouped in 19 functional categories in anaerobic cultures of EtrA7-1 compared to the wild type grown on lactate and nitrate**. The total of genes down-regulated is 323 and the up-regulated is 289. Each bar represents the number of up-regulated (white) and down-regulated (black) genes for each functional category. Abreviations: [PS], Protein synthesis; [DM], DNA Metabolism; [RF], Regulatory Function; [CIM], Central Intermediary Metabolism; [EM], Energy Metabolism; [OC], Other Categories; [UF], Unknown Function; [TBP], Transport Binding Proteins; [PF], Protein Fate; [HP], Hypothetical Protein; [AAB], Amino Acid Biosynthesis; [FAPM], Fatty Acid and Phospholipid Metabolism; [DRF], Disrupted Reading Frame; [CP], Cellular Processes; [BCPGC], Biosynthesis of Cofactors, Prosthetic Groups, and Carriers; [CE], Cell Envelope; [ST], Signal Transduction; [T], Transcription; and [PPNN], Purines, Pyrimidines, Nucleosides and Nucleotides. Click here for file

Additional file 3 **Figure SI2. Sequence logo (http://weblogo.berkeley.edu/logo.cgi) of the identified EtrA binding site motif for *S. oneidensis *MR-1**. The logo represents the palindromic model of the aligned sites, showing the relative frequency of each base at each position of the motif. The Y-axis indicates the information content measured in bits. All of the predicted sites that contribute to the model are in Table SI1 in the supplementary materials. Click here for file
