# Supplementary material for: Fnr (EtrA) acts as a fine-tuning regulator of anaerobic metabolism in Shewanella oneidensis MR-1
Source: BMC Microbiol. 2011 Mar 30;11:64. doi: 10.1186/1471-2180-11-64 (PMC3078092; doi:10.1186/1471-2180-11-64)
Supplement: Additional file 1 — Supplemental Table SI1. Genes differentially expressed in anaerobic cultures of MR-1 and Etra7-1 at different concentrations of KNO3. Complete list of genes differentially expressed including relative expression, standard deviation, "TIGR role" and predicted EtrA binding sites. [file 1471-2180-11-64-S1.PDF]

SUPPLEMENTAL TABLE SI1. Genes differentially expressed in anaerobic cultures of MR-1 and Etra7-1 at different concentrations of KNO_3.

Locus Tag	Gene name	Etra7-1 vs. MR-1 2mM KNO_3		Product Descriptor	Main role category	Subrole category	Predicted EtrA binding sites
		Relative expression [a]	STD [b]				
SO0003	trmE	2.89 [c]	1.59	tRNA modification GTPase, TrmE	Protein synthesis	tRNA and rRNA base modification	
SO0004	yidC	3.88	0.64	inner membrane insertase, YidC	Protein fate	Protein and peptide secretion and trafficking	
SO0005	yidD	7	3.12	protein of unknown function DUF37, YidD	Unknown function	General	
SO0006	rnpA	6.77	2.78	ribonuclease P protein, RnpA	Transcription	RNA processing	
SO0007	rpmH	5.03	2.9	50S ribosomal protein L34, RpmH	Protein synthesis	Ribosomal proteins: synthesis and modification	
SO0020 [d]	fadA	0.15	0.05	fatty oxidation complex, beta subunit, FadA	Fatty acid and phospholipid metabolism	Degradation	
SO0021	fadB	0.16	0.04	fatty oxidation complex, alpha subunit, FadB	Fatty acid and phospholipid metabolism	Degradation	TGTGATACACTGCACA
SO0037	rimN	2.27	0.38	threonylcarbamoyladenosine t(6)A tRNA modification protein, RimN	Protein synthesis	tRNA and rRNA base modification	
SO0095	hutI	0.5	0.14	imidazolonepropionase, HutI	Energy metabolism	Amino acids and amines	
SO0110		3.77	1.56	intramembrane peptidase, M48 family	Protein fate	Degradation of proteins, peptides, and glycopeptides	
SO0119		0.47	0.11	periplasmic protein of unknown function DUF1090	Unknown function	General	
SO0141		0.38	0.08	bifunctional diguanylate cyclase with nitrate-nitrite and PAS sensory domains/diguanylate phosphodiesterase	Regulatory functions	Small molecule interactions	

SO0142	ribB	0.41	0.11	3,4-dihydroxy-2-butanone 4-phosphate synthase, RibB	Biosynthesis of cofactors, prosthetic groups, and carriers	Riboflavin, FMN, and FAD	
SO0148		2.99	0.74	hypothetical protein	Hypothetical proteins		
SO0154		0.38	0.09	inner membrane protein of unknown function DUF893	Unknown function	General	
SO0162	pckA	2.21	0.48	phosphoenolpyruvate carboxykinase, PckA	Energy metabolism	Glycolysis/gluconeogenesis	TGTGAGCTGGATCATT
SO0163	hslO	2.82	0.45	redox regulated chaperone, HslO	Protein fate	Protein folding and stabilization	
SO0196	selD	2.64	0.57	selenophosphate synthase, SelD	Protein synthesis	tRNA aminoacylation	
SO0197	desC	2.47	0.95	stearoyl-CoA desaturase, DesC	Fatty acid and phospholipid metabolism	Biosynthesis	
SO0218	secE	3.31	1.04	preprotein translocase subunit, SecE	Protein fate	Protein and peptide secretion and trafficking	TGTGCTCAAGCTCAGG
SO0219	nusG	2.09	0.64	transcription antitermination protein, NusG	Transcription	Transcription factors	
SO0225	rpoC	2.37	1.24	DNA-directed RNA polymerase, beta-prime subunit, RpoC	Transcription	DNA-dependent RNA polymerase	
SO0230	rpsJ	2.65	0.43	ribosomal protein S10, RpsJ	Protein synthesis	Ribosomal proteins: synthesis and modification	
SO0232	rplD	2	0.62	ribosomal protein L4, RplD	Protein synthesis	Ribosomal proteins: synthesis and modification	
SO0233	rplW	2.15	0.67	ribosomal protein L23, RplW	Protein synthesis	Ribosomal proteins: synthesis and modification	
SO0241	rplN	2.48	0.49	ribosomal protein L14, RplN	Protein synthesis	Ribosomal proteins: synthesis and modification	TTTGACTAAATTCAAA
SO0242	rplX	2.89	0.59	ribosomal protein L24, RplX	Protein synthesis	Ribosomal proteins: synthesis and modification	

SO0244	rpsN	2.61	0.45	ribosomal protein S14, RpsN	Protein synthesis	Ribosomal proteins: synthesis and modification	
SO0245	rpsH	2.35	0.46	ribosomal protein S8, RpsH	Protein synthesis	Ribosomal proteins: synthesis and modification	
SO0246	rplF	2.18	0.38	ribosomal protein L6, RplF	Protein synthesis	Ribosomal proteins: synthesis and modification	
SO0249	rpmD	2.29	0.84	ribosomal protein L30, RpmD	Protein synthesis	Ribosomal proteins: synthesis and modification	
SO0250	rplO	2.19	0.57	ribosomal protein L15, RplO	Protein synthesis	Ribosomal proteins: synthesis and modification	
SO0252	rpmJ	3.82	1.59	ribosomal protein L36, RpmJ	Protein synthesis	Ribosomal proteins: synthesis and modification	
SO0255	rpsD	2.07	0.75	ribosomal protein S4, RpsD	Protein synthesis	Ribosomal proteins: synthesis and modification	
SO0258*	tnpA_ISSod26	2.19	0.9	ISSod26 transposase, TnpA_ISSod26	Mobile and extrachromosomal element functions	Transposon functions	
SO0269		0.51	0.27	cytochrome c-type biogenesis thioredoxin	Protein fate	Protein folding and stabilization	
SO0272		2.11	0.77	competence/damage-inducible protein, CinA-like	DNA metabolism	DNA replication, recombination, and repair	TGGGCACTGGTTCACA
SO0274	ppc	0.48	0.19	phosphoenolpyruvate carboxylase, Ppc	Energy metabolism	Other	
SO0286	aroK	2.21	0.48	shikimate kinase, AroK	Amino acid biosynthesis	Aromatic amino acid family	
SO0300	llpC	3.93	1.82	outer membrane lipoprotein, LppC	Cell envelope	Other	
SO0311		2.21	0.5	radical SAM superfamily protein	Unknown function	Enzymes of unknown specificity	
SO0314	speC	0.06	0.06	ornithine decarboxylase, SpeF	Central intermediary metabolism	Polyamine biosynthesis	
SO0324	ygbA	0.49	0.22	nitrosative stress induced protein, YgbA	Cellular processes	Detoxification	

SO0336		3.1	1.63	sodium:proton antiporter, NhaC family	Transport and binding proteins	Cations and iron carrying compounds	
SO0337		2.51	0.5	YER057c/Yigf/Uk114 family protein	Unknown function	General	
SO0350		0.41	0.08	conserved protein of unknown function	Unknown function	General	
SO0351		0.44	0.08	two component transcriptional regulator, LuxR family	Signal transduction	Two-component systems	
SO0361	gmk	2.13	0.44	guanylate kinase, Gmk	Purines, pyrimidines, nucleosides, and nucleotides	Nucleotide and nucleoside interconversions	
SO0393	fis	2.64	1.03	DNA-binding protein, Fis	DNA metabolism	DNA replication, recombination, and repair	
SO0394	dusB	2.35	0.6	tRNA-dihydrouridine synthase, DusB	Protein synthesis	tRNA and rRNA base modification	
SO0398	frdA	0.3	0.16	quinol:fumarate reductase, FAD-binding subunit, FrdA	Energy metabolism	Electron transport	
SO0399	frdB	0.39	0.06	quinol:fumarate reductase, FeS subunit, FrdB	Energy metabolism	Electron transport	
SO0403		0.21	0.06	conserved outer membrane protein	Cell envelope	Other	TCTGATCTAACTCATA, ATAGAGCGAATGCTCA
SO0404		0.25	0.07	zinc dependent metalloprotease domain lipoprotein	Protein fate	Degradation of proteins, peptides, and glycopeptides	
SO0429		0.29	0.13	oligopeptidase lipoprotein, M13 family	Protein fate	Degradation of proteins, peptides, and glycopeptides	
SO0433	rsd	**NDE**	**NDE**	regulator of sigma D, Rsd	Regulatory functions	Other	
SO0435	hemE	**NDE**	**NDE**	uroporphyrinogen decarboxylase, HemE	Biosynthesis of cofactors, prosthetic groups, and carriers	Heme, porphyrin, and cobalamin	
SO0438		0.39	0.26	oxidoreductase, short chain dehydrogenase/reductase family	Unknown function	Enzymes of unknown specificity	

SO0439		0.29	0.07	conserved protein of unknown function	Hypothetical proteins	Conserved	TGTGCGTCAGATCACG
SO0442	purH	2.12	0.38	bifunctional IMP cyclohydrolase/phosphoribosylaminoimidazolecarboxamide formyltransferase, PurH	Purines, pyrimidines, nucleosides, and nucleotides	Purine ribonucleotide biosynthesis	
SO0449		0.49	0.19	iron-regulated inner membrane protein	Unknown function	General	
SO0458		0.47	0.24	conserved lysine rich protein	Unknown function	General	
SO0466		1.97	0.96	protein of unknown function	Unknown function	General	
SO0487	nosF	0.28	0.06	ABC sulfur transporter, ATPase subunit, NosF	Transport and binding proteins	Cations and iron carrying compounds	
SO0488	nosY	0.42	0.2	ABC sulfur transporter, inner membrane subunit, NosY	Transport and binding proteins	Cations and iron carrying compounds	
SO0490		0.28	0.14	transmembrane transcriptional regulator	Regulatory functions	DNA interactions	
SO0491		2.03	0.27	oligopeptidase lipoprotein, M13 family	Protein fate	Degradation of proteins, peptides, and glycopeptides	
SO0516		2.96	1.18	hypothetical protein	Hypothetical proteins		
SO0520	czcA	2.08	0.34	heavy metal efflux pump, inner membrane component, CzcA	Transport and binding proteins	Cations and iron carrying compounds	
SO0541		0.5	0.14	RNA-metabolizing metallo-beta-lactamase family protein	Unknown function	Enzymes of unknown specificity	
SO0559		2.89	1.1	MaoC domain protein	Unknown function	General	
SO0560	fhs	2.14	0.46	formate--tetrahydrofolate ligase, Fhs	Central intermediary metabolism	One-carbon metabolism	
SO0567	plsC	2.63	0.55	1-acyl-sn-glycerol-3-phosphate acyltransferase, PlsC	Fatty acid and phospholipid metabolism	Biosynthesis	
SO0581		0.32	0.07	Zn-binding protein	Unknown function	General	TTTGATTTATATCAAG, GGTAATCTTAAGCACA
SO0582	tpmT	0.42	0.05	thiopurine S-methyltransferase, TpmT	Cellular processes	Detoxification	

SO0584		0.39	0.11	methyl-accepting chemotaxis protein with PAS/PAC sensory domains	Cellular processes	Chemotaxis and motility	
SO0590	psd	2.12	0.59	phosphatidylserine decarboxylase, Psd	Fatty acid and phospholipid metabolism	Biosynthesis	
SO0591	rsgA	2.58	0.9	ribosome small subunit-stimulated GTPase, RsgA	Regulatory functions	Small molecule interactions	
SO0610	petC	2.16	1.17	ubiquinol-cytochrome c reductase cytochrome c1 subunit, PetC	Energy metabolism	Electron transport	
SO0611	sspA	2.6	0.58	transcpritional activator, SspA	Cellular processes	Adaptations to atypical conditions	
SO0612	sspB	2.09	0.33	stringent starvation protein b, SspB	Cellular processes	Adaptations to atypical conditions	
SO0630	nosA	0.3	0.06	TonB-dependent outer membrane Cu receptor, NosA	Transport and binding proteins	Cations and iron carrying compounds	
SO0675		2.06	0.82	prophage MuSo1 major head subunit	Mobile and extrachromosomal element functions	Prophage functions	
SO0694	galK	0.4	0.1	galactokinase, GalK	Energy metabolism	Sugars	TTCGATTTATATCACG
SO0706		0.43	0.05	HipA family protein	Unknown function	General	
SO0708	tnpA_ISSod18	0.44	0.06	ISSod18 transposase, TnpA_ISSod18	Mobile and extrachromosomal element functions	Transposon functions	
SO0715	sorA	2.42	1.74	sulfite dehydrogenase, molybdopterin subunit, SorA	Energy metabolism	Electron transport	
SO0718		2.33	1.13	conserved hypothetical secreted protein	Hypothetical proteins	Conserved	
SO0741	ggtB	0.25	0.11	gamma-glutamyltransferase, GgtB	Biosynthesis of cofactors, prosthetic groups, and carriers	Glutathione and analogs	TTTGCTCAAGTTAAAC
SO0747	fpr	2.17	1.02	ferredoxin--NADP reductase, Fpr	Energy metabolism	Electron transport	
SO0748		4.05	1.45	conserved hypothetical protein	Hypothetical proteins	Conserved	

SO0765		0.41	0.05	threonine efflux protein, RhtB family	Transport and binding proteins	Amino acids, peptides and amines	
SO0768		5.06	2.34	NAD dependent epimerase/dehydratase family protein	Unknown function	Enzymes of unknown specificity	
SO0769	argR	5.58	2.9	arginine biosynthetic gene repressor, ArgR	Regulatory functions	DNA interactions	TTAGATCCATGTCACA
SO0777	ubiH	0.46	0.11	2-octaprenyl-6-methoxyphenol hydroxylase, UbiH	Biosynthesis of cofactors, prosthetic groups, and carriers	Menaquinone and ubiquinone	
SO0778	visC	0.32	0.11	FAD-dependent hydroxylase, VisC	Unknown function	Enzymes of unknown specificity	
SO0820	macA	0.2	0.05	macrolide eflux system, membrane fusion protein subunit, MacA	Transport and binding proteins	Other	
SO0821	macB	0.14	0.05	macrolide eflux system, ATPase and inner membrane subunit, MacB	Transport and binding proteins	Unknown substrate	
SO0822	macC	0.1	0.04	macrolide eflux system, outer membrane lipoprotein, MacC	Transport and binding proteins	Unknown substrate	
SO0827		0.31	0.07	lactate transport protein, LctP family	Transport and binding proteins	Carbohydrates, organic alcohols, and acids	TGTGCTTTAAATCGCT
SO0837	blaA	0.29	0.11	class D carbapenem-hydrolyzing beta-lactamase, BlaA	Cellular processes	Toxin production and resistance	
SO0839		0.46	0.11	transcriptional regulator, LysR family	Regulatory functions	DNA interactions	
SO0845	napB	0.15	0.04	periplasmic nitrate reductase, cytochrome c subunit, NapB	Energy metabolism	Electron transport	
SO0846	napH	0.18	0.11	quinol dehydrogenase subunit, NapH	Energy metabolism	Electron transport	
SO0847	napG	0.14	0.08	periplasmic nitrate reductase, ferredoxin component, NapG	Energy metabolism	Electron transport	
SO0848	napA	0.18	0.13	periplasmic nitrate reductase, molybdopterin-binding subunit, NapA	Energy metabolism	Electron transport	

SO0849	napD	0.3	0.04	nitrate reductase (NapA) twin-arginine signal chaperone, NapD	Protein fate	Protein and peptide secretion and trafficking	CGTGATCTAACTCTCA, GTCGATCGGGATCAAA
SO0856		0.34	0.11	ABC transporter, inner membrane protein subunit	Transport and binding proteins	Unknown substrate	
SO0859		0.45	0.15	hybrid multi-sensor histidine kinase with PAS sensory domains	Signal transduction	Two-component systems	
SO0874	dksA	1.98	1.12	RNA polymerase-binding protein, DksA	DNA metabolism	DNA replication, recombination, and repair	
SO0876	pepB	0.34	0.1	aminopeptidase, PepB	Protein fate	Degradation of proteins, peptides, and glycopeptides	
SO0882		0.37	0.12	oxidoreductase, GMC family	Unknown function	Enzymes of unknown specificity	
SO0883	gfaA	0.36	0.06	glutathione-dependent formaldehyde-activating enzyme, GfaA	Cellular processes	Detoxification	
SO0884		0.45	0.08	protein of unknown function DUF2390	Unknown function	General	
SO0903	nqrB	0.34	0.15	Na-translocating NADH-quinone reductase subunit B, NqrB	Energy metabolism	Electron transport	TTTGCTGTAAAGCAAA, TGTGCATGGAATCGCC
SO0904	nqrC	0.28	0.09	Na-translocating NADH-quinone reductase subunit C, NqrC	Energy metabolism	Electron transport	
SO0905	nqrD	0.27	0.14	Na-translocating NADH-quinone reductase subunit D, NqrD	Energy metabolism	Electron transport	
SO0906	nqrE	0.23	0.07	Na-translocating NADH-quinone reductase subunit E, NqrE	Energy metabolism	Electron transport	
SO0907	nqrF	0.23	0.09	Na-translocating NADH-quinone reductase subunit F, NqrF	Energy metabolism	Electron transport	
SO0908		0.31	0.13	ankyrin domain protein	Unknown function	General	
SO0935	nhaD	0.18	0.11	sodium:proton antiporter, NhaD	Transport and binding proteins	Cations and iron carrying compounds	TGTGATTGCTATCAAC
SO0936		0.22	0.06	transmembrane transcriptional regulator	Regulatory functions	DNA interactions	ATTGATGTAATTCACA

SO0940		0.36	0.08	transmembrane transcriptional regulator	Regulatory functions	DNA interactions	TGTGAGCGAAGGCACG, TGTGAACTAAAGCAGA, CGGGATCACAGTAATC
SO0941		0.33	0.05	hypothetical periplasmic protein	Hypothetical proteins		
SO0944		0.35	0.07	radical SAM superfamily protein	Unknown function	Enzymes of unknown specificity	
SO0970	fccA	0.31	0.17	periplasmic fumarate reductase, FccA	Energy metabolism	Electron transport	
SO0975		0.37	0.06	conserved hypothetical inner membrane protein	Hypothetical proteins	Conserved	
SO1018	nuoE	0.44	0.17	NADH-ubiquinone oxidoreductase subunit E, NuoE	Energy metabolism	Electron transport	
SO1019	nuoCD	0.35	0.13	NADH-ubiquinone oxidoreductase subunit CD, NuoCD	Energy metabolism	Electron transport	
SO1020	nuoB	0.4	0.1	NADH-ubiquinone oxidoreductase subunit B, NuoB	Energy metabolism	Electron transport	
SO1036	cobS	0.42	0.05	cobalamin 5-phosphate synthase, CobS	Biosynthesis of cofactors, prosthetic groups, and carriers	Heme, porphyrin, and cobalamin	
SO1037	cobU	0.36	0.05	bifunctional cobinamide kinase/cobinamide phosphate guanylyltransferase, CobU	Biosynthesis of cofactors, prosthetic groups, and carriers	Heme, porphyrin, and cobalamin	
SO1038	cobQ	0.5	0.19	cobyric acid synthase, CobQ	Biosynthesis of cofactors, prosthetic groups, and carriers	Heme, porphyrin, and cobalamin	
SO1047	lrgA	0.27	0.04	inner membrane protein, LrgA	Cell envelope	Biosynthesis and degradation of murein sacculus and peptidoglycan	
SO1048	lrgB	0.23	0.02	inner membrane protein, LrgB	Cell envelope	Biosynthesis and degradation of murein sacculus and peptidoglycan	
SO1049		0.47	0.13	GCN5-related N-acetyltransferase	Unknown function	Enzymes of unknown specificity	

SO1075		0.27	0.14	trypsin-like serine and cysteine peptidase	Protein fate	Degradation of proteins, peptides, and glycopeptides	
SO1103	nqrA	2.25	0.54	Na-translocating NADH-quinone reductase subunit A, NqrA	Energy metabolism	Electron transport	CGTGATTGCGATCGCA, TCTGCGCTAGCTCAAT
SO1104	nqrB	2.7	1.02	Na-translocating NADH-quinone reductase subunit B, NqrB	Energy metabolism	Electron transport	
SO1105	nqrC	3.15	0.8	Na-translocating NADH-quinone reductase subunit C, NqrC	Energy metabolism	Electron transport	
SO1106	nqrD	4.65	2.07	Na-translocating NADH-quinone reductase subunit D, NqrD	Energy metabolism	Electron transport	
SO1107	nqrE	3.63	1.62	Na-translocating NADH-quinone reductase subunit E, NqrE	Energy metabolism	Electron transport	
SO1108	nqrF	4.21	2.05	Na-translocating NADH-quinone reductase subunit F, NqrF	Energy metabolism	Electron transport	
SO1109	apbE	6.44	2.17	Fe-S assembly/repair lipoprotein, ApbE	Biosynthesis of cofactors, prosthetic groups, and carriers	Other	TGTGAATTAGCCCAAG, TTTGCATTAAAGGGCA
SO1121	proB	0.43	0.12	glutamate 5-kinase, ProB	Amino acid biosynthesis	Glutamate family	
SO1122	proA	0.33	0.08	gamma-glutamyl phosphate reductase, ProA	Amino acid biosynthesis	Glutamate family	
SO1135		0.48	0.1	conserved hypothetical protein	Hypothetical protein	Conserved	
SO1163	ybeD	2.24	0.61	protein lipolyation system protein, YbeD	Biosynthesis of cofactors, prosthetic groups, and carriers	Lipoate	
SO1164	dacA	2.67	0.69	D-alanyl-D-alanine carboxypeptidase, DacA	Cell envelope	Biosynthesis and degradation of surface polysaccharides and lipopolysaccharides	

SO1166	mltB	2.03	0.92	lytic murein transglycosylase B, MltB	Cell envelope	Biosynthesis and degradation of surface polysaccharides and lipopolysaccharides	
SO1167	rodA	2.93	0.69	rod shape-determining protein, RodA	Cell envelope	Biosynthesis and degradation of surface polysaccharides and lipopolysaccharides	
SO1168	mrdA	2.94	1.02	penicillin-binding protein 2, MrdA	Cell envelope	Biosynthesis and degradation of surface polysaccharides and lipopolysaccharides	
SO1169	rlmH	3.37	0.26	rRNA large subunit methyltransferase H, RlmH	Protein synthesis	tRNA and rRNA base modification	
SO1170		2.14	1	protein of unknown function DUF143	Unknown function	General	
SO1193	secD	6.4	2.28	protein-export membrane protein, SecD	Protein fate	Protein and peptide secretion and trafficking	
SO1194	secF	6.25	0.91	protein-export membrane protein, SecF	Protein fate	Protein and peptide secretion and trafficking	
SO1202		2.02	0.44	protein of unknown function DUF150	Unknown function	General	
SO1203	nusA	2.5	0.36	N utilization substance protein A, NusA	Transcription	Transcription factors	
SO1205	rbfA	4.34	1.57	ribosome-binding factor A, RbfA	Transcription	RNA processing	
SO1206	truB	3.17	0.61	tRNA pseudouridine synthase B, TruB	Protein synthesis	tRNA and rRNA base modification	
SO1207	rpsO	2.83	1.53	ribosomal protein S15, RpsO	Protein synthesis	Ribosomal proteins: synthesis and modification	
SO1218	deoA	0.51	0.22	thymidine phosphorylase, DeoA	Purines, pyrimidines, nucleosides, and nucleotides	Other	TGTGACCTAAATCTAG
SO1221	deoD	0.38	0.26	purine nucleoside phosphorylase, DeoD	Purines, pyrimidines, nucleosides, and nucleotides	Salvage of nucleosides and nucleotides	
SO1245		5.26	0.92	integral inner membrane protein	Unknown function	General	TTTCATTCAACTCAAA, TGTGAGCGAGATAACA

SO1265	puuR	2.04	0.87	putrescine degradation gene regulator, PuuR	Regulatory functions	DNA interactions	
SO1276	puuE	2.04	0.88	GABA aminotransferase, PLP-dependent, PuuE	Central intermediary metabolism	Other	
SO1277		0.45	0.29	proton:oligopeptide symporter, POT family	Transport and binding proteins	Unknown substrate	TGGGAGCGTCATCACA, AATGAACTAGATCAAA
SO1278		0.37	0.09	methyl-accepting chemotaxis protein	Cellular processes	Chemotaxis and motility	
SO1284	rpoD	2.7	1.23	RNA polymerase sigma-70 factor, RpoD	Transcription	Transcription factors	
SO1286	dnaG	2.75	1.06	DNA primase, DnaG	DNA metabolism	DNA replication, recombination, and repair	
SO1288	rpsU	3.63	2.82	ribosomal protein S21, RpsU	Protein synthesis	Ribosomal proteins: synthesis and modification	
SO1337		2.13	0.7	conserved periplasmic protein	Hypothetical protein	Conserved	
SO1347	lepB	3.47	0.36	signal peptidase I, LepB	Protein fate	Protein and peptide secretion and trafficking	
SO1348	rnc	3.02	0.89	ribonuclease III, Rnc	Transcription	RNA processing	
SO1349	era	2.7	0.51	GTP-binding protein, Era	Regulatory functions	Other	
SO1350	recO	5	1.77	DNA repair protein, RecO	DNA metabolism	DNA replication, recombination, and repair	
SO1351	pdxJ	3.96	0.75	pyridoxal phosphate biosynthetic protein, PdxJ	Biosynthesis of cofactors, prosthetic groups, and carriers	Pyridoxine	
SO1357	rpsP	2.41	0.96	ribosomal protein S16, RpsP	Protein synthesis	Ribosomal proteins: synthesis and modification	
SO1358	rimM	2.08	0.38	16S rRNA processing protein, RimM	Transcription	RNA processing	
SO1360	rplS	2.19	0.25	ribosomal protein L19, RplS	Protein synthesis	Ribosomal proteins: synthesis and modification	
SO1363	hcp	0.13	0.08	hydroxylamine reductase, Hcp	Cellular processes	Detoxification	

SO1364	hcr	0.12	0.07	NADH oxidoreductase, Hcr	Cellular processes	Detoxification	
SO1365		0.12	0.05	conserved hypothetical inner membrane protein	Hypothetical protein	Conserved	
SO1366		0.16	0.02	sodium:proton antiporter, CPA1 family	Transport and binding proteins	Cations and iron carrying compounds	
SO1383		3.84	1.44	ATP-dependent RNA helicase, DEAD box family	Transcription	Other	
SO1408		0.42	0.14	helicase	Transcription	Other	
SO1424		2.3	0.53	predicted outer membrane lipoprotein	Cell envelope	Other	
SO1428	dmsF	0.31	0.05	outer membrane protein, DmsF	Protein fate	Protein and peptide secretion and trafficking	TGTGATACAATTCAAA
SO1429	dmaA	0.43	0.09	surface localized dimethyl sulfoxide reductase, molybdopterin-binding subunit, DmsA	Energy metabolism	Electron transport	
SO1430	dmsB	0.29	0.04	dimethyl sulfoxide reductase, FeS subunit, DmsB	Energy metabolism	Electron transport	
SO1431	dsmG	0.32	0.05	DMSO reductase chaperone/maturation protein, DmsG	Protein fate	Protein and peptide secretion and trafficking	
SO1432	dsmH	0.25	0.07	conserved hypothetical cytoplasmic protein, DmsH	Protein fate	Protein folding and stabilization	
SO1457	hsdM	0.46	0.09	type I restriction-modification system, M subunit, HsdM	DNA metabolism	DNA replication, recombination, and repair	
SO1463*		2.51	0.37	hypothetical protein with C-terminal P-loop ATPase domain	Hypothetical proteins	Domain	
SO1476	bamE	3.17	2.09	beta barrel protein translocation component, BamE	Protein fate	Protein and peptide secretion and trafficking	
SO1490	adhB	0.28	0.12	alcohol dehydrogenase II, AdhB	Energy metabolism	Fermentation	TGTGATCTAGATCGGT, TTGGAACTAGATAACT
SO1495	glgX	2.03	0.5	glycogen isoamylase, GlgX	Energy metabolism	Biosynthesis and degradation of polysaccharides	

SO1498	glgC	6.86	4.9	glucose-1-phosphate adenylyltransferase, GlgC	Energy metabolism	Biosynthesis and degradation of polysaccharides	
SO1499	glgA	5.42	5.26	glycogen synthase, GlgA	Energy metabolism	Biosynthesis and degradation of polysaccharides	
SO1513		0.4	0.04	conserved lipoprotein of unknown function	Cell envelope	Other	
SO1518	lldG	0.21	0.07	L-lactate dehydrogenase complex protein, LldG	Energy metabolism	Electron transport	
SO1519	lldF	0.15	0.06	L-lactate dehydrogenase, iron-sulfur cluster-binding protein, LldF	Energy metabolism	Electron transport	
SO1520	lldE	0.25	0.07	L-lactate dehydrogenase complex protein, LldE	Energy metabolism	Electron transport	
SO1522		0.47	0.07	lactate transport protein, LctP family	Transport and binding proteins	Carbohydrates, organic alcohols, and acids	TGTGATTCATATCACT, TGTGATCGATATCACT
SO1536	rlmM	2.18	0.9	2 -O-ribose methyltransferase, RlmM	Protein synthesis	tRNA and rRNA base modification	
SO1539		0.51	0.12	periplasmic peptidase, family S9	Protein fate	Degradation of proteins, peptides, and glycopeptides	
SO1558	phoB	5.22	2.59	two component transcriptional regulator for the phosphate regulon, PhoB	Signal transduction	Two-component systems	
SO1559	phoR	3.68	1.86	sensor histidine kinase for the phosphate regulon with PAS sensory domain, PhoR	Signal transduction	Two-component systems	
SO1560		4.51	3.46	phosphate-binding protein	Regulatory functions	Other	
SO1561		0.35	0.22	peptidase, M1 family	Protein fate	Degradation of proteins, peptides, and glycopeptides	
SO1617		3.17	0.77	conserved hypothetical protein	Hypothetical proteins	Conserved	
SO1618		3.34	0.97	conserved protein of unknown function	Hypothetical proteins	Conserved	
SO1619		2.18	0.86	protein of unknown function DUF446	Unknown function	General	

SO1631	pyrH	2.98	0.72	uridylate kinase, PyrH	Purines, pyrimidines, nucleosides, and nucleotides	Nucleotide and nucleoside interconversions	
SO1632	frr	2.64	0.46	ribosome recycling factor, Frr	Protein synthesis	Translation factors	
SO1633	uppS	2.81	0.59	undecaprenyl diphosphate synthase, UppS	Biosynthesis of cofactors, prosthetic groups, and carriers	Other	
SO1634	cdsA	2.29	0.49	phosphatidate cytidylyltransferase, CdsA	Fatty acid and phospholipid metabolism	Biosynthesis	
SO1635	dxr	2.12	0.57	1-deoxy-D-xylulose 5-phosphate reductoisomerase, Dxr	Biosynthesis of cofactors, prosthetic groups, and carriers	Other	
SO1636	rseP	2.6	0.28	intramembrane zinc metalloprotease, RseP	Protein fate	Degradation of proteins, peptides, and glycopeptides	
SO1648	cspG	2.46	1.75	cold shock protein, CspG	Cellular processes	Adaptations to atypical conditions	
SO1664	galE	0.45	0.15	UDP-glucose 4-epimerase, GalE	Energy metabolism	Sugars	
SO1669	tyrR	0.34	0.13	aromatic amino acid biosynthesis/transport regulator, TyrR	Regulatory functions	Protein interactions	
SO1673	ompW	0.28	0.08	outer membrane protein, OmpW	Cell envelope	Other	CACGCGCTCGCTCACA, CGTGATTTGGATCTCA, TTTGATTTGGATCAAT
SO1676	metA	0.36	0.13	homoserine O-succinyltransferase, MetA	Amino acid biosynthesis	Aspartate family	
SO1682	mmsB	2.06	0.91	3-hydroxyisobutyrate dehydrogenase, MmsB	Energy metabolism	Amino acids and amines	
SO1686		2.88	0.37	peptidase S9, prolyl oligopeptidase lipoprotein	Protein fate	Degradation of proteins, peptides, and glycopeptides	
SO1690		3.01	0.94	ABC transporter, ATPase subunit	Transport and binding proteins	Unknown substrate	
SO1695		0.39	0.32	diguanylate cyclase with PAS sensory domain	Regulatory functions	Small molecule interactions	

SO1723		7.86	3.76	ABC phosphate transporter, inner membrane subunit, PstC-like	Transport and binding proteins	Anions	
SO1724		4.75	2.78	ABC phosphate transporter, inner membrane subunit, PstA-like	Transport and binding proteins	Anions	
SO1725		3.56	2.75	ABC phosphate transporter, ATPase subunit, PstB-like	Transport and binding proteins	Anions	
SO1726	phoU	2.59	1.77	phosphate transport system regulatory protein, PhoU	Regulatory functions	Other	
SO1776	mtrB	0.22	0.04	outer membrane protein, MtrB	Protein fate	Protein and peptide secretion and trafficking	
SO1777	mtrA	0.25	0.06	periplasmic decaheme cytochrome c, MtrA	Energy metabolism	Electron transport	
SO1778	mtrC	0.3	0.09	surface localized decaheme cytochrome c surface lipoprotein, MtrC	Energy metabolism	Electron transport	
SO1779	omcA	0.3	0.05	surface localized decaheme cytochrome c, OmcA	Energy metabolism	Electron transport	GTGGAATTAGATCCCA, TGTGATTGAGATCTGA, TTTGAGGTAGATAACA
SO1807	pspA	3.11	1.64	phage shock protein A, PspA	Cellular processes	Adaptations to atypical conditions	
SO1808	pspB	3.49	1.42	phage shock protein B, PspB	Cellular processes	Adaptations to atypical conditions	
SO1809	pspC	3.45	1.84	phage shock protein C, PspC	Cellular processes	Adaptations to atypical conditions	
SO1831		2.2	0.54	putative outer membrane lipoprotein of unknown function DUF885	Cell envelope	Other	
SO1854		2.08	0.52	secreted protein	Unknown function	General	
SO1860	uvrY	0.41	0.1	two component transcriptional regulator, UvrY	Signal transduction	Two-component systems	
SO1861	uvrC	0.29	0.06	excinuclease ABC, C subunit, UvrC	DNA metabolism	DNA replication, recombination, and repair	

SO1875	tnpA_ISSod4	0.44	0.07	ISSod4 transposase, TnpA_ISSod4	Mobile and extrachromosomal element functions	Transposon functions	
SO1881		0.35	0.07	type I secretion system, membrane fusion protein, RND family	Transport and binding proteins	Unknown substrate	TGTTAGCTCGGTCAAA
SO1882		0.26	0.12	type I secretion system, inner membrane component, RND superfamily	Transport and binding proteins	Unknown substrate	
SO1888		0.45	0.1	conserved hypothetical protein	Hypothetical proteins	Conserved	
SO1891	scoB	3.77	1.8	acetyl-CoA:acetoacetate-CoA transferase, alpha subunit, AtoA	Energy metabolism	Other	
SO1892	scoA	3.21	2.14	acetyl-CoA:acetoacetate-CoA transferase, beta subunit, AtoD	Energy metabolism	Other	
SO1899		0.44	0.12	periplasmic protein with peptidoglycin binding domain	Unknown function	General	
SO1900	prpE	0.32	0.08	propionyl-CoA synthetase, PrpE	Fatty acid and phospholipid metabolism	Biosynthesis	
SO1911		0.25	0.17	oxidoreductase, short chain dehydrogenase/reductase family	Unknown function	Enzymes of unknown specificity	AGTGAGCACTATCTAA, TTTGAATTGGCGCACA
SO1924		2.53	1.27	cation efflux protein, AcrB/AcrD/AcrF family	Transport and binding proteins	Cations and iron carrying compounds	
SO1925		2.68	1.43	type I secretion system, membrane fusion protein, RND family	Transport and binding proteins	Other	
SO1927	sdhC	2.47	1.27	succinate dehydrogenase, cytochrome b556 subunit, SdhC	Energy metabolism	TCA cycle	
SO1930	sucA	3.02	1.22	2-oxoglutarate dehydrogenase, E1 component, SucA	Energy metabolism	TCA cycle	

SO1931	sucB	3.6	1.59	succinyl-CoA: dihydrolipoate S-succinyltransferase, E2 component, SucB	Energy metabolism	TCA cycle	
SO1932	sucC	3.29	0.98	succinyl-CoA synthase, beta subunit, SucC	Energy metabolism	TCA cycle	
SO1933	sucD	3.28	1.24	succinyl-CoA synthase, alpha subunit, SucD	Energy metabolism	TCA cycle	
SO1983*		4.96	1.49	conserved hypothetical protein	Hypothetical proteins	Conserved	
SO1984		4.04	1.43	hypothetical protein	Hypothetical proteins		TTTGATACAAATCAAA
SO1997		0.38	0.15	family M1 unassigned peptidases	Protein fate	Degradation of proteins, peptides, and glycopeptides	
SO2002		2.14	0.85	conserved secreted protein	Unknown function	General	
SO2006		2.36	0.5	tRNA-dihydrouridine synthase	Protein synthesis	tRNA and rRNA base modification	
SO2012	apt	2.17	0.36	adenine phosphoribosyltransferase , Apt	Purines, pyrimidines, nucleosides, and nucleotides	Salvage of nucleosides and nucleotides	TTTGATCTAAGGCACA
SO2016	htpG	0.46	0.17	heat shock protein, HtpG	Protein fate	Protein folding and stabilization	
SO2017		0.45	0.26	heat shock response protein	Cellular processes	Adaptations to atypical conditions	
SO2019	hemH	0.41	0.26	ferrochelatase, HemH	Biosynthesis of cofactors, prosthetic groups, and carriers	Heme, porphyrin, and cobalamin	
SO2042	yedY	0.42	0.1	periplasmic oxidoreductase, molybdenum-binding subunit, YedY	Energy metabolism	Electron transport	
SO2043	yedZ	0.42	0.1	oxidoreductase, inner membrane cytochrome b subunit , YedZ	Energy metabolism	Electron transport	
SO2045		0.45	0.08	cation efflux protein, CDF family	Transport and binding proteins	Cations and iron carrying compounds	TGTGATCTAGGTCACC, GTAGCGCTTGCTCACA
SO2046		0.46	0.11	transcriptional regulator, MarR family	Regulatory functions	DNA interactions	

SO2090	hypE	0.32	0.06	NiFe hydrogenase accessory/formation protein, HypE	Protein fate	Protein folding and stabilization	
SO2093	hypB	0.33	0.15	NiFe hydrogenase nickel incorporation-associated protein, HypB	Protein fate	Protein folding and stabilization	
SO2094	hypF	0.19	0.09	NiFe hydrogenase assembly protein, HypF	Protein fate	Protein modification and repair	
SO2095	hyaE	0.11	0.09	NiFe hydrogenase assembly chaperone, HyaE	Protein fate	Protein modification and repair	
SO2097	hyaC	0.07	0.04	NiFe hydrogenase, cytochrome b subunit, HyaC	Energy metabolism	Electron transport	
SO2098	hyaB	0.11	0.1	NiFe hydrogenase, large subunit, HyaB	Energy metabolism	Electron transport	
SO2099	hyaA	0.07	0.11	NiFe hydrogenase, small subunit, HyaA	Energy metabolism	Electron transport	
SO2103		0.37	0.14	cytoplasmic ApbE family protein	Unknown function	General	
SO2109		0.39	0.08	peptidoglycan-binding protein of unknown function	Unknown function	General	
SO2110		4.13	1.52	secreted protein of unknown function DUF882	Unknown function	General	CTTGATCTGGATAGCA
SO2112	rplY	2.28	1.56	ribosomal protein L25, RplY	Protein synthesis	Ribosomal proteins: synthesis and modification	TGTGATCAAGATCACG, TTTGATTCAGATCAAA
SO2114		0.45	0.11	surface antigen	Cell envelope	Other	
SO2136	adhE	0.4	0.1	aldehyde-alcohol dehydrogenase, AdhE	Energy metabolism	Fermentation	
SO2149	recC	0.49	0.06	exodeoxyribonuclease V, gamma subunit, RecC	DNA metabolism	DNA replication, recombination, and repair	
SO2153		0.46	0.07	AAA+ ATPase, MoxR MRP family	Unknown function	Enzymes of unknown specificity	
SO2178	ccpA	0.24	0.1	diheme cytochrome c5 peroxidase, CcpA	Cellular processes	Detoxification	TGTGAGCTAATTCGCT, TGTCATTCATTTCAAA
SO2191	metC	0.43	0.11	cystathionine beta-lyase, MetC	Amino acid biosynthesis	Aspartate family	

SO2192		0.28	0.19	periplasmic sensor histidine kinase	Signal transduction	Two-component systems	
SO2194		0.37	0.23	MotB family protein	Unknown function	General	
SO2195		0.32	0.16	inter-alpha-trypsin inhibitor family protein	Regulatory functions		
SO2196	srtA	0.46	0.24	sortase, SrtA	Protein fate	Protein and peptide secretion and trafficking	
SO2198		3.38	1.65	peptidase, M28 family	Protein fate	Degradation of proteins, peptides, and glycopeptides	
SO2199		2.71	0.58	porin protein	Transport and binding proteins	Porins	
SO2213	algA	0.43	0.1	alpha-glucosidase, AlgA	Energy metabolism	Sugars	
SO2214*	gluP	0.41	0.1	glucose permease, GluP	Transport and binding proteins	Carbohydrates, organic alcohols, and acids	
SO2228		0.19	0.03	CBS domain protein	Unknown function	General	
SO2251		0.37	0.1	protein of unknown function UPF0227	Unknown function	General	
SO2260	suhB	3.26	1.81	inositol-phosphate phosphatase, SuhB	Cell envelope	Biosynthesis and degradation of murein sacculus and peptidoglycan	
SO2261	trmJ	2.04	0.5	tRNA:Cm32/Um32 methyltransferase, TrmJ	Protein synthesis	tRNA and rRNA base modification	
SO2264	iscS	0.42	0.27	cysteine desulfurase, IscS	Biosynthesis of cofactors, prosthetic groups, and carriers	Other	
SO2280		0.29	0.12	multidrug efflux protein, MFS superfamily	Transport and binding proteins	Other	
SO2300	infC	2.14	0.99	translation initiation factor IF-3, InfC	Protein synthesis	Translation factors	
SO2350	aspC	0.46	0.13	aspartate aminotransferase, AspC	Amino acid biosynthesis	Aspartate family	
SO2352		3.11	0.93	beta-D-glucosaminidase	Cell envelope	Biosynthesis and degradation of murein sacculus and peptidoglycan	
SO2354	ydaO	3.29	0.69	tRNA(Cytosine32)-2-thiocytidine synthetase,YdaO	Protein synthesis	tRNA and rRNA base modification	

SO2355	uspE	2.3	0.31	universal stress protein, UspE	Cellular processes	Adaptations to atypical conditions	ATTGTTCTAGATCAAA
SO2356	etrA	0.05	0.01	oxygen sensitive electron transport regulator A, EtrA	Regulatory functions	DNA interactions	
SO2361	ccoP	2.3	0.92	Cbb3-type cytochrome c oxidase, subunit III, CcoP	Energy metabolism	Electron transport	
SO2362	ccoQ	3.44	1.16	Cbb3-type cytochrome c oxidase, subunit IV, CcoQ	Energy metabolism	Electron transport	
SO2364	ccoN	2.76	1.08	Cbb3-type cytochrome c oxidase, subunit I, CcoN	Energy metabolism	Electron transport	CTTGAGCCATGTCAAA, GTTGATCTAGATCAAT
SO2366		0.42	0.22	metal-dependent phosphohydrolase with response regulator receiver modulation	Signal transduction	Two-component systems	
SO2378	tusB	2.27	0.54	tRNA 2-thiouridine synthesizing protein, B subunit, TusB	Protein synthesis	tRNA and rRNA base modification	
SO2395		0.13	0.03	acyl-CoA dehydrogenase family protein	Fatty acid and phospholipid metabolism	Degradation	
SO2419	fadH	0.17	0.05	NADP-dependent 2,4-dienoyl-CoA reductase, FadH	Fatty acid and phospholipid metabolism	Degradation	
SO2420	sppA	0.32	0.05	protease IV, SppA	Protein fate	Degradation of proteins, peptides, and glycopeptides	
SO2426		3.06	1.47	two component transcriptional regulator, Winged helix family	Signal transduction	Two-component systems	
SO2469		0.32	0.06	TonB-dependent receptor	Transport and binding proteins	Unknown substrate	ATTGATGAAGATCACA
SO2471	dapE	0.45	0.09	succinyl-diaminopimelate desuccinylase, DapE	Amino acid biosynthesis	Aspartate family	TGCGAGCAAAATCAAT
SO2472		0.36	0.05	D-alanyl-D-alanine carboxypeptidase-related protein	Unknown function	Enzymes of unknown specificity	
SO2473		0.38	0.08	hydrolase, alpha/beta fold family	Unknown function	Enzymes of unknown specificity	

SO2485	dgt	0.47	0.12	deoxyguanosinetriphosphate triphosphohydrolase, Dgt	Purines, pyrimidines, nucleosides, and nucleotides	Nucleotide and nucleoside interconversions	
SO2492		0.09	0.04	oxidoreductase, acyl-CoA dehydrogenase family	Unknown function	Enzymes of unknown specificity	
SO2493	psrA	0.14	0.03	fatty acid degradation transcriptional regulator, PsrA	Regulatory functions	DNA interactions	
SO2497		0.43	0.13	conserved hypothetical SEC-C domain protein	Hypothetical proteins	Conserved	
SO2501		1.98	0.45	radical activating enzyme	Protein fate	Protein modification and repair	
SO2504		0.4	0.06	intramembrane peptidase, rhomboid family	Protein fate	Degradation of proteins, peptides, and glycopeptides	
SO2536	fadE	0.17	0.06	acyl-CoA dehydrogenase, FadE	Fatty acid and phospholipid metabolism	Degradation	TGTGATCGCAATCAAC
SO2538		0.4	0.11	response regulator	Signal transduction	Two-component systems	
SO2539		0.43	0.1	cyclic diguanylate phosphodiesterase with response regulator receiver modulation and PAS sensing domain	Signal transduction	Two-component systems	
SO2542		0.48	0.08	signaling protein with FIST domain	Signal transduction		
SO2566	asmA	0.47	0.12	outer membrane protein assembly protein, AsmA	Protein fate	Protein and peptide secretion and trafficking	
SO2567		0.34	0.07	Rra-like regulator of RNAse E	Transcription	Transcription factors	
SO2569		0.41	0.19	protein of unknown function	Unknown function	General	
SO2570		0.15	0.06	conserved lipoprotein of unknown function DUF885	Cell envelope	Other	
SO2587	hemB	0.36	0.2	delta-aminolevulinic acid dehydratase, HemB	Biosynthesis of cofactors, prosthetic groups, and carriers	Heme, porphyrin, and cobalamin	TGTGATCGCTATCACT

SO2601	prc	0.39	0.09	periplasmic C-terminal processing protease, Prc	Protein fate	Degradation of proteins, peptides, and glycopeptides	
SO2610	ycfH	2.44	0.62	TatD-related deoxyribonuclease, YcfH	Unknown function	Enzymes of unknown specificity	TGGGATCGAGGTCAAA
SO2636		2.63	0.56	cupin family protein	Unknown function	General	
SO2639		0.5	0.17	hypothetical protein	Hypothetical proteins		
SO2684	I	2.54	0.3	prophage MuSo2 protease, I	Mobile and extrachromosomal element functions	Prophage functions	
SO2685	T	2.23	0.64	prophage MuSo2 major head subunit, T	Mobile and extrachromosomal element functions	Prophage functions	
SO2687		2.04	0.57	prophage MuSo2 hypothetical protein	Mobile and extrachromosomal element functions	Prophage functions	
SO2702	J	3.16	0.69	prophage MuSo2 baseplate assembly protein, J	Mobile and extrachromosomal element functions	Prophage functions	
SO2715		1.98	0.58	TonB-dependent receptor	Transport and binding proteins	Unknown substrate	
SO2727	cctA	0.32	0.23	periplasmic tetraheme cytochrome c, CctA	Energy metabolism	Electron transport	
SO2736		2.98	2.04	conserved hypothetical outer membrane protein	Hypothetical proteins	Conserved	
SO2750	tolR	2.95	1.07	TonB system biopolymer transport inner membrane component, TolR	Transport and binding proteins	Other	
SO2759	upp	0.28	0.14	uracil phosphoribosyltransferase, Upp	Purines, pyrimidines, nucleosides, and nucleotides	Salvage of nucleosides and nucleotides	CGTGAGCAATATCCCA
SO2768		0.37	0.07	acyl-CoA dehydrogenase family protein	Fatty acid and phospholipid metabolism	Degradation	
SO2771	garR	0.31	0.08	2-hydroxy-3-oxopropionate reductase, GarR	Energy metabolism	Amino acids and amines	

SO2774	fabF	3.07	0.27	3-oxoacyl-(acyl-carrier-protein) synthase II , FabF	Fatty acid and phospholipid metabolism	Biosynthesis	
SO2777	fabD	3.35	0.61	malonyl CoA-acyl carrier protein transacylase, FabD	Fatty acid and phospholipid metabolism	Biosynthesis	
SO2778	fabH	2.14	0.29	3-oxoacyl-(acyl-carrier-protein) synthase III, FabH	Fatty acid and phospholipid metabolism	Biosynthesis	
SO2780	rpmF	2.05	1.44	ribosomal protein L32, RpmF	Protein synthesis	Ribosomal proteins: synthesis and modification	
SO2786*		2.43	0.99	sulfate transporter	Transport and binding proteins	Cations and iron carrying compounds	
SO2791	cdd	0.38	0.12	cytidine deaminase, Cdd	Purines, pyrimidines, nucleosides, and nucleotides	Salvage of nucleosides and nucleotides	CGCGAACTAACTCGCA
SO2792		0.48	0.1	protein of unknown function DUF1289	Unknown function	General	
SO2796		0.37	0.13	dual DUF748 domain protein	Unknown function	General	
SO2806		0.26	0.03	phospahte starvation inducible E-like protein	Cellular processes	Adaptations to atypical conditions	TGTGAATTGAGTCACA
SO2815		0.34	0.14	transporter-like protein, HCC family	Transport and binding proteins	Unknown substrate	
SO2820*		0.14	0.04	ISSod19 transposase, TnpA_ISSod19	Mobile and extrachromosomal element functions	Transposon functions	TGTGAATCCATTCACA
SO2821	yecH	0.21	0.16	protein of unknown function, YecH	Unknown function	General	CCTGATCTTGTGCACA
SO2823		0.41	0.03	two component transcriptional regulator	Signal transduction	Two-component systems	CCTGATCTTGTGCACA
SO2882	prkA	0.48	0.14	serine protein kinase, PrkA	Unknown function	Enzymes of unknown specificity	
SO2883		0.4	0.13	protein of unknown function DUF444	Unknown function	General	
SO2886	nhaB	0.27	0.08	sodium:proton antiporter, NhaB	Transport and binding proteins	Cations and iron carrying compounds	
SO2887	dsbB	6.14	3.76	disulfide bond formation protein, DsbB	Protein fate	Protein modification and repair	

SO2889		2.57	0.53	multi-sensor histidine kinase with Cache and PAS sensory domains	Signal transduction	Two-component systems	
SO2898	smc	0.5	0.09	chromosome partition protein, Smc	Cellular processes	Cell division	GTTGATCACAATCGCA
SO2899	cysZ	0.47	0.13	protein of unknown function DUF540, CysZ	Unknown function	General	GTTGATCACAATCGCA
SO2905		0.45	0.1	O-methyltransferase	Unknown function	Enzymes of unknown specificity	
SO2907		0.32	0.13	TonB-dependent receptor	Transport and binding proteins	Unknown substrate	
SO2911	focA	0.4	0.2	bidirectional formate transporter, FocA	Transport and binding proteins	Carbohydrates, organic alcohols, and acids	TTTGAGCTGAAACAAA
SO2912	pflB	0.18	0.11	pyruvate formate-lyase, PflB	Energy metabolism	Fermentation	TTTGAGCTGAAACAAA
SO2913	pflA	0.2	0.13	pyruvate formate-lyase 1 activating enzyme, PflA	Energy metabolism	Fermentation	
SO2914		0.22	0.13	protein of unknown function DUF412	Unknown function	General	
SO2915	ackA	0.24	0.16	acetate kinase, AckA	Central intermediaty metabolism	Other	
SO2916	pta	0.23	0.14	phosphate acetyltransferase, Pta	Energy metabolism	Electron transport	
SO2924		3.07	2.51	signal peptidase I	Protein fate	Protein and peptide secretion and trafficking	
SO2940	J	3.58	1.09	prophage LambdaSo tail fiber protein, J	Mobile and extrachromosomal element functions	Prophage functions	
SO2941	I	2.7	0.72	prophage LambdaSo tail assembly protein, I	Mobile and extrachromosomal element functions	Prophage functions	
SO2943*		0.32	0.11	prophage LambdaSo tail assembly protein	Mobile and extrachr	Prophage functions	
SO2944		2.49	1.25	prophage LambdaSo structural protein	Mobile and extrachromosomal element functions	Prophage functions	
SO2945		3.14	0.89	prophage LambdaSo tail fiber protein	Mobile and extrachromosomal element functions	Prophage functions	

SO2946		2.88	0.95	prophage LambdaSo protein with carbohydrate-binding module	Mobile and extrachromosomal element functions	Prophage functions	
SO2948	K	2.53	0.44	prophage LambdaSo tail assembly protein, K	Mobile and extrachromosomal element functions	Prophage functions	
SO2951		2.41	0.77	prophage LambdaSo protein	Mobile and extrachromosomal element functions	Prophage functions	
SO2953	H	3.61	1.02	prophage LambdaSo tail length tape meausure protein, H	Mobile and extrachromosomal element functions	Prophage functions	
SO2954		2.2	1.32	prophage LambdaSo hypothetical protein	Mobile and extrachromosomal element functions	Prophage functions	
SO2955	G	3.81	0.84	prophage LambdaSo minor tail protein, G	Mobile and extrachromosomal element functions	Prophage functions	
SO2956	V	2.67	1.47	prophage LambdaSo major tail protein, V	Mobile and extrachromosomal element functions	Prophage functions	
SO2957		3.69	1.27	prophage LambdaSo conserved hypothetical protein	Mobile and extrachromosomal element functions	Prophage functions	
SO2958		3.9	1.3	prophage LambdaSo conserved hypothetical protein	Mobile and extrachromosomal element functions	Prophage functions	
SO2960		3.51	0.63	prophage LambdaSo phage head-tail adaptor	Mobile and extrachromosomal element functions	Prophage functions	
SO2961		2.64	0.57	prophage LambdaSo conserved hypothetical protein	Mobile and extrachromosomal element functions	Prophage functions	
SO2962		4.86	0.97	prophage LambdaSo helical domain protein	Mobile and extrachromosomal element functions	Prophage functions	TGTGACTAAAACCAAA
SO2963		4.57	0.65	prophage LambdaSo major capsid protein	Mobile and extrachromosomal element functions	Prophage functions	

SO2964		6.17	0.95	prophage LambdaSo head maturation protease	Mobile and extrachromosomal element functions	Prophage functions	
SO2965	B	7.02	0.93	prophage LambdaSo portal protein, B	Mobile and extrachromosomal element functions	Prophage functions	
SO2967	A	6.01	0.69	prophage LambdaSo terminase, A	Mobile and extrachr	Prophage functions	
SO2968	A	4.38	1.13	prophage LambdaSo terminase, A	Mobile and extrachromosomal element functions	Prophage functions	
SO2969		5.6	1.27	prophage LambdaSo endonuclease, HNH family	Mobile and extrachromosomal element functions	Prophage functions	
SO2970		4.92	1.54	prophage LambdaSo hypothetical protein	Mobile and extrachromosomal element functions	Prophage functions	
SO2971		3.56	0.85	prophage LambdaSo conserved hypothetical protein	Mobile and extrachromosomal element functions	Prophage functions	
SO2972		5.31	1.67	prophage LambdaSo conserved hypothetical protein	Mobile and extrachromosomal element functions	Prophage functions	
SO2973	R	4.43	0.93	prophage LambdaSo lysozyme, R	Mobile and extrachromosomal element functions	Prophage functions	
SO2974		2.1	0.82	prophage LambdaSo pyridoxal phosphate dependent enzyme	Mobile and extrachromosomal element functions	Prophage functions	
SO2990	cI	0.43	0.16	prophage LambdaSo lytic gene repressor, CI	Mobile and extrachromosomal element functions	Prophage functions	
SO3019	trpE	0.17	0.1	anthranilate synthase component I, TrpE	Amino acid biosynthesis	Aromatic amino acid family	
SO3020	trpG	0.12	0.04	anthranilate synthase component II, glutamine amido-transferase, TrpG	Amino acid biosynthesis	Aromatic amino acid family	
SO3021	trpD	0.12	0.04	anthranilate phosphoribosyltransferase , TrpD	Amino acid biosynthesis	Aromatic amino acid family	

SO3022	trpC/F	0.14	0.06	bifunctional indole-3-glycerolphosphate synthetase/phosphoribosylanthranilate isomerase, TrpCF	Amino acid biosynthesis	Aromatic amino acid family	
SO3023	trpB	0.22	0.05	tryptophan synthase, beta subunit, TrpB	Amino acid biosynthesis	Aromatic amino acid family	
SO3024	trpA	0.26	0.08	tryptophan synthase, alpha subunit, TrpA	Amino acid biosynthesis	Aromatic amino acid family	
SO3025		0.44	0.14	periplasmic esterase	Unknown function	Enzymes of unknown specificity	
SO3059		0.34	0.18	sigma54 specific transcriptional regulator, Fis family	Regulatory functions	Protein interactions	TTTGATTGCAGGCACA
SO3069	fimV	0.44	0.14	type IV pilus assembly protein, FimV	Protein fate	Protein and peptide secretion and trafficking	
SO3072	fabB	2.44	0.88	3-oxoacyl-(acyl-carrier-protein) synthase I, FabB	Fatty acid and phospholipid metabolism	Biosynthesis	
SO3083		0.37	0.15	peptidase, subfamily M16A	Protein fate	Degradation of proteins, peptides, and glycopeptides	
SO3088	fadJ	0.32	0.18	anaerobic fatty oxidation complex, alpha subunit, FadJ	Fatty acid and phospholipid metabolism	Degradation	
SO3089	fadI	0.26	0.05	anaerobic fatty oxidation complex, beta subunit, FadI	Fatty acid and phospholipid metabolism	Degradation	TGTGATCTGATTCTAA
SO3094	batB	0.46	0.17	TPR domain protein, BatB	Unknown function	General	
SO3095	batD	0.44	0.07	protein of unknown function, BatD	Unknown function	General	
SO3096		0.5	0.14	RNA polymerase sigma-70 factor, ECF subfamily	Transcription	Transcription factors	
SO3099		4.43	1.24	outer membrane long-chain fatty acid transport protein, FadL-family	Transport and binding proteins	Carbohydrates, organic alcohols, and acids	
SO3108		0.51	0.09	bifunctional 1,3-dimethyluroporphyrinogen III dehydrogenase/siroheme ferrochelatase	Biosynthesis of cofactors, prosthetic groups, and carriers	Heme, porphyrin, and cobalamin	

SO3117		0.41	0.11	thioredoxin	Energy metabolism	Electron transport	
SO3119		0.43	0.22	conserved hypothetical protein	Hypothetical proteins	Conserved	CGCGATCTTTATCACT
SO3120		0.23	0.13	oxidoreductase, Gfo/Idh/MocA family (NAD binding)	Energy metabolism	Electron transport	CGCGATCTTTATCACT
SO3121		0.4	0.2	conserved hypothetical protein	Hypothetical proteins	Conserved	
SO3142	dcp	0.36	0.21	peptidyl-dipeptidase, Dcp	Protein fate	Degradation of proteins, peptides, and glycopeptides	
SO3144	etfA	0.36	0.13	electron transfer flavoprotein, alpha subunit, EtfA	Energy metabolism	Electron transport	
SO3148		0.29	0.11	periplasmic amidohydrolase family protein	Unknown function	Enzymes of unknown specificity	
SO3149		0.26	0.11	periplasmic amidohydrolase family protein	Unknown function	Enzymes of unknown specificity	
SO3151		0.4	0.08	protein of unknown function UPF0066	Unknown function	General	
SO3152		0.42	0.04	acetyltransferase, CysE/LacA/LpxA/NodL family	Unknown function	Enzymes of unknown specificity	
SO3172	wbfU	2.31	0.65	galactosyl transferase, WbfU	Cell envelope	Biosynthesis and degradation of surface polysaccharides and lipopolysaccharides	
SO3176	wbpT	2.01	0.88	O-antigen biosynthesis glycosyltransferase, family 4	Cell envelope	Biosynthesis and degradation of surface polysaccharides and lipopolysaccharides	
SO3177		2.1	0.79	formyltransferase domain protein	Unknown function	Enzymes of unknown specificity	
SO3178		2.19	0.93	polysaccharide deacetylase	Cell envelope	Biosynthesis and degradation of surface polysaccharides and lipopolysaccharides	

SO3179	wzy	2.63	1.66	O-antigen polymerase, Wzy	Cell envelope	Biosynthesis and degradation of surface polysaccharides and lipopolysaccharides	
SO3270	pseC	0.49	0.09	C4 aminotransferase for PseB product, PseC	Cell envelope	Biosynthesis and degradation of surface polysaccharides and lipopolysaccharides	
SO3284	ybgT	0.22	0.05	conserved hypothetical protein, YbgT	Hypothetical proteins	Conserved	
SO3285	cydB	0.21	0.06	cytochrome d ubiquinol oxidase, subunit II, CydB	Energy metabolism	Electron transport	
SO3286	cydA	0.22	0.1	cytochrome d ubiquinol oxidase, subunit I, CydA	Energy metabolism	Electron transport	TTTGATTCAAATCAAT
SO3293	guaB	2.15	0.55	inosine-5-monophosphate dehydrogenase, GuaB	Purines, pyrimidines, nucleosides, and nucleotides	Purine ribonucleotide biosynthesis	
SO3308	der	2.22	0.64	ribosome biogenesis GTP-binding protein, Der	Protein synthesis	Ribosomal proteins: synthesis and modification	
SO3315	rlmN	2.68	0.46	23S rRNA methyltransferase, RlmN	Protein synthesis	tRNA and rRNA base modification	
SO3361		0.34	0.18	periplasmic protein of unknown function DUF885	Unknown function	General	
SO3364		3.68	1.63	secreted protein of unknown function	Unknown function	General	
SO3389		0.42	0.11	bifunctional signalling protein with PAS and GGDEF domains/diguanylate phosphodiesterase	Regulatory functions	Small molecule interactions	
SO3391		0.5	0.09	ATP-dependent protease, family S16 unassigned peptidases	Protein fate	Degradation of proteins, peptides, and glycopeptides	
SO3395		2.58	1.2	conserved hypothetical protein	Hypothetical proteins	Conserved	
SO3411		0.25	0.15	subfamily S41B non-peptidase homologues	Protein fate	Degradation of proteins, peptides, and glycopeptides	

SO3420		0.32	0.14	monoheme cytochrome c-prime	Energy metabolism	Electron transport	
SO3483		0.22	0.08	type I secretion system, membrane fusion protein, RND family	Transport and binding proteins	Other	TGTTAGCCGATTCAAA
SO3484		0.16	0.07	type I secretion system, inner membrane component, RND superfamily	Transport and binding proteins	Other	
SO3542	xfp	0.3	0.24	D-xylulose 5-phosphate/D-fructose 6-phosphate phosphoketolase, Xfp	Energy metabolism		
SO3553		0.27	0.14	sulfate transporter, SulP family	Transport and binding proteins	Anions	
SO3563		0.33	0.11	SM-20 domain protein	Unknown function	General	
SO3564	dcp	0.26	0.17	peptidyl-dipeptidase Dcp	Protein fate	Degradation of proteins, peptides, and glycopeptides	
SO3582		2.95	0.52	methyl-accepting chemotaxis protein	Cellular processes	Chemotaxis and motility	
SO3645		2.7	0.27	conserved hypothetical secreted protein	Hypothetical proteins	Conserved	
SO3646	folA	4.06	1.47	dihydrofolate reductase, FolA	Biosynthesis of cofactors, prosthetic groups, and carriers	Folic acid	
SO3647		4.5	2.19	conserved hypothetical inner membrane protein	Hypothetical proteins	Conserved	
SO3648		4.14	0.8	conserved hypothetical inner membrane protein, DUF1212-like	Hypothetical proteins	Conserved	
SO3649	cgtA	2.26	1.09	regulator of ppGpp phosphohydrolase, CgtA	Regulatory functions		
SO3652	rplU	2.71	1.04	ribosomal protein L21, RplU	Protein synthesis	Ribosomal proteins: synthesis and modification	
SO3677		0.4	0.04	hypothetical protein	Hypothetical proteins		
SO3678		0.33	0.16	FAD-dependent oxidoreductase	Unknown function	Enzymes of unknown specificity	

SO3684		0.31	0.24	transcriptional regulator, TetR family	Regulatory functions	DNA interactions	CGTGATCTTGCTCATA, CTTGCACAAAATCACG
SO3741	pntB	0.51	0.19	NAD(P) transhydrogenase, beta subunit, PntB	Energy metabolism	Electron transport	TGTGCACTGGTTAAAA, TTTGTTTTAATGCACA
SO3747	cvrA	2.16	0.58	sodium:proton antiporter, CvrA	Transport and binding proteins	Cations and iron carrying compounds	
SO3748	ybiS	2.05	0.48	L,D-transpeptidase, YbiS	Cell envelope	Biosynthesis and degradation of murein sacculus and peptidoglycan	
SO3770		3.16	1.58	phosphate transport accessory protein of unknown function DUF47	Transport and binding proteins	Anions	
SO3776		0.49	0.05	conserved hypothetical inner membrane protein	Hypothetical proteins	Conserved	
SO3778	cyaB	0.35	0.08	cytoplasmic adenylate cyclase, CyaB	Regulatory functions	Small molecule interactions	
SO3779	cydC	0.44	0.12	ABC glutathione transporter, ATPase/inner membrane subunit, CydC	Transport and binding proteins	Other	
SO3780	cydD	0.32	0.08	ABC glutathione transporter, ATPase/inner membrane subunit, CydD	Transport and binding proteins	Other	
SO3783		2.55	0.94	ATP-dependent RNA helicase, DEAD box family	Transcription	Other	
SO3792	sufC	0.52	0.18	FeS assembly ATPase, SufC	Biosynthesis of cofactors, prosthetic groups, and carriers	Other	
SO3820*	amtB	0.16	0.26	ammonium transporter, AmtB	Transport and binding proteins	Cations and iron carrying compounds	
SO3829		2.23	0.27	conserved hypothetical protein	Hypothetical proteins	Conserved	
SO3830		2.76	0.89	SirB family protein	Unknown function	General	
SO3888		0.47	0.16	tandem DUF1508 repeat protein of unknown function	Unknown function	General	
SO3891		0.2	0.11	conserved hypothetical protein	Hypothetical proteins	Conserved	TTTGAACCAGATCACA, ATTGATCAGGATCAGT

SO3896	omp35	0.26	0.17	outer membrane porin, Omp35	Transport and binding proteins	Porins	
SO3898	sndH	0.46	0.1	periplasmic quinoprotein dehydrogenase	Energy metabolism	Sugars	
SO3899	parE	0.48	0.08	DNA topoisomerase IV, B subunit, ParE	DNA metabolism	DNA replication, recombination, and repair	
SO3900		0.3	0.16	enzyme of unknown function UPF0227	Unknown function	Enzymes of unknown specificity	
SO3901	cpdA	0.45	0.26	cyclic 3-prime,5-prime-adenosine monophosphate phosphodiesterase, CpdA	Regulatory functions	Other	
SO3909		2.37	1.11	conserved hypothetical inner membrane protein	Hypothetical proteins	Conserved	
SO3912	dusA	4.2	0.99	tRNA-dihydrouridine synthase, DusA	Protein synthesis	tRNA and rRNA base modification	
SO3928	rpsR	2.18	0.65	ribosomal protein S18, RpsR	Protein synthesis	Ribosomal proteins: synthesis and modification	
SO3929	priB	3.27	1.07	primosomal replication protein N, PriB	DNA metabolism	DNA replication, recombination, and repair	
SO3933		2.11	0.76	transport protein, MFS superfamily	Transport and binding proteins	Unknown substrate	
SO3934	rlmB	3.53	1.47	23S rRNA methyltransferase, RlmB	Protein synthesis	tRNA and rRNA base modification	
SO3980	nrfA	0.18	0.06	ammonia-forming nitrite reductase, NrfA	Energy metabolism	Electron transport	TTTGCGCTAGATCAAA
SO3985		2.26	0.61	succinylglutamate desuccinylase/aspartoacylase family protein	Unknown function	Enzymes of unknown specificity	
SO3986	lysC	2.86	1	aspartokinase III, lysine-sensitive, LysC	Amino acid biosynthesis	Aspartate family	
SO3993		0.43	0.31	hypothetical protein	Hypothetical proteins		
SO4001*		0.41	0.08	hybrid histidine kinase/chemotaxis response CheY regulator receiver	Signal transduction		

SO4002		0.46	0.11	multi-sensor histidine kinase with PAS and small molecule binding domains	Signal transduction	Two-component systems	
SO4008		2.11	0.35	protein of unknown function	Unknown function	General	
SO4011		2.75	1.58	conserved hypothetical protein	Hypothetical proteins	Conserved	
SO4034	deaD	2.51	0.36	ATP-dependent RNA helicase, DeaD	Transcription	Other	
SO4035		4.96	1.23	hypothetical protein	Hypothetical proteins		
SO4038		0.44	0.24	protein of unknown function	Unknown function	General	
SO4055	metL	0.45	0.1	bifunctional aspartokinase II/homoserine dehydrogenase, methionine-sensitive, MetL	Amino acid biosynthesis	Aspartate family	
SO4120	rpmE	2.35	0.6	ribosomal protein L31, RpmE	Protein synthesis	Ribosomal proteins: synthesis and modification	
SO4136	speC	2.01	0.6	ornithine decarboxylase, SpeC	Central intermediary metabolism	Polyamine biosynthesis	
SO4145		2.08	0.34	signalling protein with EAL and C2 domains	Regulatory functions	Small molecule interactions	
SO4205		0.52	0.28	periplasmic protein	Unknown function	General	
SO4207		0.54	0.26	diguanylate cyclase	Regulatory functions	Small molecule interactions	
SO4208	hemB	0.44	0.15	delta-aminolevulinic acid dehydratase, HemB	Biosynthesis of cofactors, prosthetic groups, and carriers	Heme, porphyrin, and cobalamin	
SO4210*	tnpA_ISSod31	3.3	1.9	ISSod31 transposase, TnpA_ISSod31	Mobile and extrachromosomal element functions	Transposon functions	
SO4213		2.33	1.07	conserved hypothetical protein	Hypothetical proteins	Conserved	
SO4226	ftsL	0.48	0.06	cell division protein, FtsL	Cellular processes	Cell division	

SO4246	rpmG	2.63	1.54	ribosomal protein L33, RpmG	Protein synthesis	Ribosomal proteins: synthesis and modification	
SO4254	folE	2.77	0.55	GTP cyclohydrolase I, FolE	Biosynthesis of cofactors, prosthetic groups, and carriers	Folic acid	
SO4289	pstB	2.08	1.02	ABC phosphate transporter, ATPase subunit, PstB	Transport and binding proteins	Anions	
SO4290	pstA	2.04	1.07	ABC phosphate transporter, inner membrane subunit, PstA	Transport and binding proteins	Anions	
SO4299	catB	0.5	0.06	chloramphenicol O-acetyltransferase, CatB	Cellular processes	Toxin production and resistance	
SO4302	dnrN	0.25	0.14	repair of iron centers (RIC) protein, DnrN	Protein fate	Protein modification and repair	
SO4312	cyaA	2.11	0.69	adenylate cyclase, CyaA	Regulatory functions	Small molecule interactions	TGTGCTCATGTTAACA, TGAGAGGTCCATCACA, TTAGCATTGGCGCAAC
SO4317		2.55	0.63	cell surface calcium-binding protein with BNR repeats	Cellular processes	Pathogenesis	
SO4318		4.16	1.31	type I secretion system, ATPase/C39 family cysteine peptidase component	Protein fate	Protein and peptide secretion and trafficking	
SO4319		6.21	1.58	type I secretion system, membrane fusion protein, HlyD family	Protein fate	Protein and peptide secretion and trafficking	
SO4320	aggA	7.18	2.36	type I secretion system, outer membrane component, AggA	Protein fate	Protein and peptide secretion and trafficking	
SO4321		7.1	1.77	OmpA family lipoprotein	Cell envelope	Other	
SO4322		7.24	2.46	conserved hypothetical periplasmic protein	Hypothetical proteins	Conserved	
SO4323		6.91	2.29	signalling protein with GGDEF and EAL domains	Regulatory functions	Small molecule interactions	
SO4343	agxT	2.38	1.78	serine-pyruvate aminotransferase, AgxT	Central intermediary metabolism	One-carbon metabolism	

SO4344	ilvA	2.57	0.59	threonine dehydratase, IlvA	Amino acid biosynthesis	Pyruvate family	
SO4347	ilvG	0.45	0.2	acetolactate synthase II, large subunit, IlvG	Amino acid biosynthesis	Pyruvate family	
SO4349	ilvC	0.33	0.22	ketol-acid reductoisomerase, IlvC	Amino acid biosynthesis	Pyruvate family	TGTGATTGAAGTCACT
SO4371		2.06	0.93	AMP-dependent synthetase and ligase	Fatty acid and phospholipid metabolism	Biosynthesis	
SO4372		2.1	0.59	thioester dehydrase family protein	Fatty acid and phospholipid metabolism	Biosynthesis	
SO4376		2.36	0.94	conserved periplasmic protein in fatty acid biosynthesis locus	Fatty acid and phospholipid metabolism	Biosynthesis	
SO4377		2.56	0.89	inner membrane protein in fatty acid biosynthesis locus	Fatty acid and phospholipid metabolism	Biosynthesis	
SO4378		2.65	1.51	FAD-binding protein	Fatty acid and phospholipid metabolism	Biosynthesis	
SO4380		2.77	1.2	beta-ketoacyl synthase	Fatty acid and phospholipid metabolism	Biosynthesis	
SO4381		2.31	0.98	thioester dehydrase family protein	Fatty acid and phospholipid metabolism	Biosynthesis	
SO4382	fabG	2.13	0.51	3-oxoacyl-(acyl-carrier-protein) reductase, FabG	Fatty acid and phospholipid metabolism	Biosynthesis	
SO4383	fabF	2.09	0.99	3-oxoacyl-(acyl-carrier-protein) synthase II, FabF	Fatty acid and phospholipid metabolism	Biosynthesis	
SO4384		2.87	0.75	conserved periplasmic protein in fatty acid biosyntheis locus	Fatty acid and phospholipid metabolism	Biosynthesis	
SO4385		0.41	0.11	von Willebrand factor type A domain protein	Unknown function	General	
SO4400		0.41	0.12	prolyl aminopeptidase, S33 family	Protein fate	Degradation of proteins, peptides, and glycopeptides	TTTATTCAGATAACA, AGCGCGTCGGATCAAA, CATGAGCAGATGCTAA

SO4404		0.24	0.1	iron-sulfur cluster-binding protein	Energy metabolism	Electron transport	TGTGAGCCATAGCGCG, TTGGATCTATCGCACA
SO4405	katG	0.31	0.09	catalase/peroxidase HPI, KatG	Cellular processes	Detoxification	ATTGATTTTAATCAGA
SO4408	bipA	2.45	0.38	GTP-binding protein, BipA	Regulatory functions	Other	
SO4425		2.94	1.65	diguanylate cyclase	Regulatory functions	Small molecule interactions	
SO4427		2.01	0.48	periplasmic sensor histidine kinase	Signal transduction	Two-component systems	
SO4446		0.24	0.12	ABC molybdenum transporter, ATPase subunit, ModC-like	Transport and binding proteins	Anions	
SO4447		0.32	0.2	ABC molybdenum transporter, inner membrane subunit, ModB-like	Transport and binding proteins	Anions	
SO4448		0.35	0.17	ABC molybdenum transporter, periplasmic ligand-binding subunit, ModA-like	Transport and binding proteins	Anions	
SO4449	moaE	0.35	0.2	molybdenum cofactor biosynthesis protein E, MoaE	Biosynthesis of cofactors, prosthetic groups, and carriers	Molybdopterin	
SO4451	moaC	0.31	0.08	molybdenum cofactor biosynthesis protein C, MoaC	Biosynthesis of cofactors, prosthetic groups, and carriers	Molybdopterin	
SO4452	moaA	0.53	0.21	molybdenum cofactor biosynthesis protein A, MoaA	Biosynthesis of cofactors, prosthetic groups, and carriers	Molybdopterin	
SO4453		0.4	0.13	electron transfer flavoprotein-ubiquinone oxidoreductase, EtfQ	Energy metabolism	Electron transport	
SO4468	ydhM	0.46	0.13	transcriptional regulator, YdhM	Regulatory functions	DNA interactions	
SO4474*		0.2	0.03	hypothetical protein	Hypothetical proteins		
SO4475	fieF	0.3	0.1	cadmium and zinc efflux system protein, FieF	Transport and binding proteins	Cations and iron carrying compounds	

SO4476	cpxP	0.14	0.1	periplasmic stress adaptor protein, CpxP	Regulatory functions	Other	
SO4477	cpxR	0.32	0.1	two component transcriptional regulator for periplasmic stress, CpxR	Signal transduction	Two-component systems	
SO4478	cpxA	0.34	0.15	periplasmic sensor histidine kinase, CpxA	Signal transduction	Two-component systems	
SO4487		2.17	0.48	two component transcriptional regulator, Winged helix family	Signal transduction	Two-component systems	
SO4488		3.27	1.61	periplasmic sensor histidine kinase	Signal transduction	Two-component systems	
SO4489		3.76	0.91	GCN5-related N-acetyltransferase	Unknown function	Enzymes of unknown specificity	
SO4490		2.66	0.97	hypothetical protein	Hypothetical proteins		
SO4508		2.26	1.01	formate dehydrogenase accessory protein	Protein fate	Protein folding and stabilization	
SO4509	fdhA	2.33	0.56	formate dehydrogenase, molybdopterin-binding subunit, FdhA	Energy metabolism	Electron transport	
SO4510	fdhB	4.03	1.57	formate dehydrogenase, FeS subunit, FdhB	Energy metabolism	Electron transport	
SO4511	fdhC	2.53	0.32	formate dehydrogenase, cytochrome b subunit, FdhC	Energy metabolism	Electron transport	
SO4512		0.1	0.02	formate dehydrogenase accessory protein	Protein fate	Protein folding and stabilization	ACTGTTCTAGATCAAA
SO4513	fdhA	0.06	0.02	formate dehydrogenase, molybdopterin-binding subunit, FdhA	Energy metabolism	Electron transport	
SO4515	fdhC	0.07	0.01	formate dehydrogenase, cytochrome b subunit, FdhC	Energy metabolism	Electron transport	
SO4537		0.32	0.19	serine peptidase, family S9	Protein fate	Degradation of proteins, peptides, and glycopeptides	
SO4538		0.28	0.15	peptidase, M16 family	Protein fate	Degradation of proteins, peptides, and glycopeptides	

SO4559		0.54	0.29	Sel1 domain-containing protein	Hypothetical proteins	Domain	
SO4561		2.14	0.29	conserved protein of unknown function	Unknown function	General	
SO4591	cymA	0.39	0.27	membrane anchored tetraheme cytochrome c, CymA	Energy metabolism	Electron transport	
SO4596	czcC	2.39	1.35	heavy metal efflux pump ATPase subunit, CzcC	Transport and binding proteins	Cations and iron carrying compounds	
SO4597	czcB	2.47	1.56	heavy metal efflux pump, membrane fusion protein, CzcB	Transport and binding proteins	Cations and iron carrying compounds	
SO4598	czcA	2.82	1.86	heavy metal efflux pump, inner membrane component, CzcA	Transport and binding proteins	Cations and iron carrying compounds	
SO4603	lexA	0.47	0.12	SOS-response repressor and protease, LexA	Regulatory functions	DNA interactions	
SO4626	bioH	4.42	1.43	biotin biosynthesis carboxylesterase, BioH	Biosynthesis of cofactors, prosthetic groups, and carriers	Biotin	
SO4628		5.25	1.66	sulfatase	Unknown function	Enzymes of unknown specificity	
SO4633	ompR	0.38	0.19	two component transcriptional regulator for porins, OmpR	Signal transduction	Two-component systems	
SO4634	envZ	0.33	0.2	periplasmic sensor histidine kinase for osmolarity response, EnvZ	Signal transduction	Two-component systems	
SO4713	menF	0.34	0.14	isochorismate synthase, menaquinone-specific, MenF	Biosynthesis of cofactors, prosthetic groups, and carriers	Menaquinone and ubiquinone	
SO4714		0.24	0.04	conserved hypothetical protein	Hypothetical proteins	Conserved	
SO4716		0.34	0.09	GCN5-related N-acetyltransferase	Unknown function	Enzymes of unknown specificity	
SO4722	mobA	0.47	0.07	tungstopterin-guanine dinucleotide biosynthesis protein A, MobA	Biosynthesis of cofactors, prosthetic groups, and carriers	Molybdopterin	TGAGATCGAGCTCACA, TTTGCTTTCAACCGAA

SO4723		0.36	0.06	bifunctional tungstopterin-guanine dinucleotide biosynthesis protein	Biosynthesis of cofactors, prosthetic groups, and carriers	Molybdopterin	
SO4724		0.3	0.07	tungsten cofactor biosynthesis protein	Biosynthesis of cofactors, prosthetic groups, and carriers	Molybdopterin	
SO4728		2.38	0.83	protein of unknown function DUF414	Unknown function	General	
SO4729		3.84	1.91	protein of unknown function DUF2489	Unknown function	General	
SOA0001	repA	0.03	0.01	plasmid replication protein, RepA	DNA metabolism	DNA replication, recombination, and repair	
SOA0003		0.03	0.01	type II restriction endonuclease, subtype P	DNA metabolism	Restriction/modification	
SOA0004		0.02	0.02	type II DNA modification methyltransferase, subtype gamma	DNA metabolism	Restriction/modification	
SOA0019	tnpR_ISSod9	0.32	0.07	ISSod9 resolvase, TnpR_ISSod9	Mobile and extrachromosomal element functions	Transposon functions	
SOA0058		2.7	0.74	hypothetical protein	Hypothetical proteins		
SOA0060		0.1	0.02	acetyltransferase, GCN5 family	Unknown function	Enzymes of unknown specificity	
SOA0061		0.08	0.02	plasmid stability/partitioning determinant	Mobile and extrachromosomal element functions	Plasmid functions	
SOA0086		3	0.94	ISSpu16 resolvase	Mobile and extrachromosomal element functions	Transposon functions	
SOA0106		0.47	0.13	methyl-accepting chemotaxis protein	Cellular processes	Chemotaxis and motility	
SOA0123*	tnpA_ISSod37	0.48	0.18	ISSod37 transposase, TnpA_ISSod37	Mobile and extrachromosomal element functions	Transposon functions	
SOA0153	czcA	2.2	0.65	heavy metal efflux pump, inner membrane component, CzcA	Transport and binding proteins	Cations and iron carrying compounds	

SOA0157		0.29	0.11	hypothetical periplasmic protein	Hypothetical proteins		
SOA0158		0.44	0.18	hypothetical periplasmic protein	Hypothetical proteins		
SOA0172		0.07	0.03	site-specific recombinase, resolvase family	DNA metabolism	DNA replication, recombination, and repair	TATGATTTAATTCGAA, TTCGAACAAAATCACG

[a] The relative expression is presented as the ratio of the dye intensity of the anaerobic cultures with 2 mM KNO_3 of EtrA7-1 to that of MR-1 (reference).

[b] The standard deviation was calculated from six data points, which included three independent biological samples and two technical samples for each biological sample.

[c] Cells in red represent down-regulation of the gene, while cells in green represent up-regulation of the gene in EtrA 7-1 in relation to MR-1 (reference).

[d] Rows highlighted represent operons with a predicted EtrA binding site motif.

NDE - Not Differentially Expressed

* Pseudogene
