# Supplementary figures and images for: Fnr (EtrA) acts as a fine-tuning regulator of anaerobic metabolism in Shewanella oneidensis MR-1
Source: BMC Microbiol. 2011 Mar 30;11:64. doi: 10.1186/1471-2180-11-64 (PMC3078092; doi:10.1186/1471-2180-11-64)

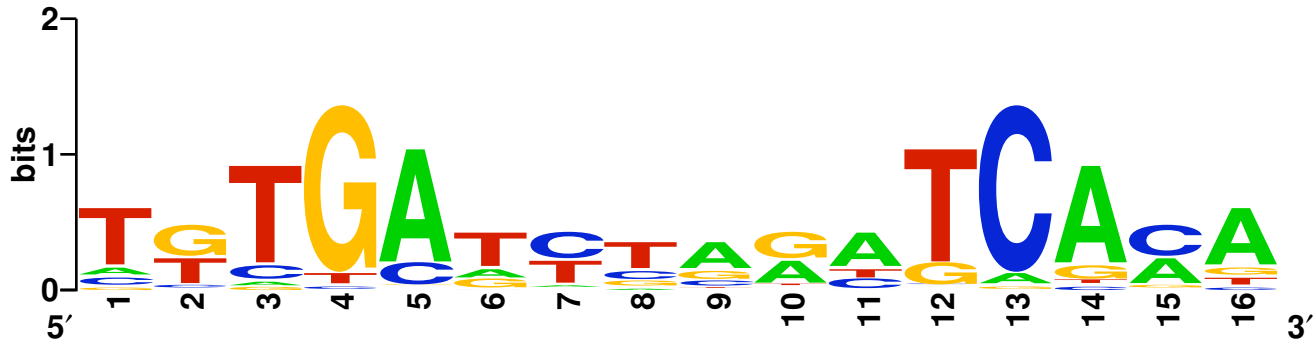

Supplement: Additional file 3 — Figure SI2. Sequence logo (http://weblogo.berkeley.edu/logo.cgi) of the identified EtrA binding site motif for S. oneidensis MR-1. The logo represents the palindromic model of the aligned sites, showing the relative frequency of each base at each position of the motif. The Y-axis indicates the information content measured in bits. All of the predicted sites that contribute to the model are in Table SI1 in the supplementary materials. [file 1471-2180-11-64-S3.PDF]
