# Supplementary material for: Fnr (EtrA) acts as a fine-tuning regulator of anaerobic metabolism in Shewanella oneidensis MR-1
Source: BMC Microbiol. 2011 Mar 30;11:64. doi: 10.1186/1471-2180-11-64 (PMC3078092; doi:10.1186/1471-2180-11-64)
Supplement: Supplementary file 3 [file 1471-2180-11-64-S2.DOC]

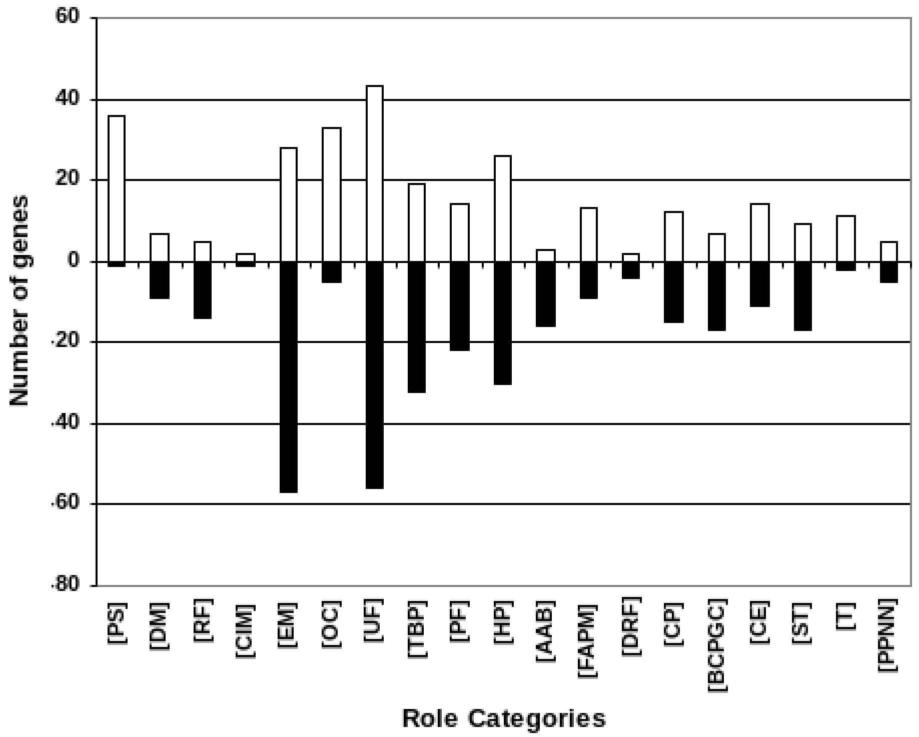

60
40
20
0
-20
-40
-60
-80
Number of genes
[PS]
[DM]
[RF]
[CIM]
[EM]
[OC]
[UF]
[TBP]
[PF]
[HP]
[AAB]
[FAPM]
[DRF]
[CP]
[BCPGC]
[CE]
[ST]
[T]
[PPNN]
Role Categories
